# Socioeconomic disparities in the association between macroeconomic changes and suicide: a population-level time series study in Australia

**DOI:** 10.1016/j.lanwpc.2026.101927

**Published:** 2026-07-18

**Authors:** Phillip Cheuk Fung Law, Lennart Reifels, Paul Corcoran, Mei Dong, Sarantis Tsiaplias, Sangsoo Shin, Jane Pirkis, Gregory Armstrong, Vikas Arya, Keith Hawton, Mark Sinyor, Eve Griffin, Lay San Too

**Affiliations:** a Centre for Mental Health and Community Wellbeing, Melbourne School of Population and Global Health, The University of Melbourne, Parkville, Victoria, Australia; b National Suicide Research Foundation, University College Cork, Cork, Ireland; c School of Public Health, University College Cork, Cork, Ireland; d Department of Economics, The University of Melbourne, Parkville, Victoria, Australia; e Melbourne Institute of Applied Economic and Social Research, The University of Melbourne, Parkville, Victoria, Australia; f Centre for Suicide Research, University of Oxford, Oxford, UK; g Department of Psychiatry, University of Toronto, Toronto, Ontario, Canada; h Department of Psychiatry, Sunnybrook Health Sciences Centre, Toronto, Ontario, Canada

**Keywords:** Suicide, Macroeconomic, Inflation, Interest rate, Savings, Debt, Economic crisis, Cost-of-living, Financial hardship, Australia

## Abstract

**Background:**

In the current cost-of-living crisis, there is concern that sharp rises in annual inflation and the benchmark interest rate may increase suicide, particularly for vulnerable groups. We aimed to examine the association between relevant macroeconomic changes and suicide across the socioeconomic spectrum to inform policy responses.

**Methods:**

In this population-level time series study, we obtained monthly suicide counts, annual inflation, and the cash rate from January 2006 to October 2023 for Australia. We also obtained related indictors, quarterly household saving to income ratio and owner-occupier housing debt to income ratio, for the same period. We conducted Poisson regression analysis at the monthly/quarterly level to examine the associations between these macroeconomic indicators (lagged by one month/quarter) and suicide for all persons and by five socioeconomic groups (Quintile 1 being the most disadvantaged to Quintile 5 being the least disadvantaged).

**Findings:**

In Australia, every 1% monthly increase in annual inflation was associated with a 4% increase in suicide rate one month later in the general population [Incidence Rate Ratio (IRR) 1.04, 95% Confidence Interval (CI) 1.01‒1.06, p = 0.011]. The increase was greater (7%) for people residing in the most and the least disadvantaged areas. For the latter group, such increase persisted three months later. A 1% quarterly increase in household saving to income ratio was associated with a 1% increase in suicide rate one quarter later among people residing in the least disadvantaged areas (Quintile 5: IRR 1.01, 95% CI 1.00–1.02, p = 0.019). A 1% quarterly increase in housing debt to income ratio was associated with a 6% increase in suicide rate in the same quarter among people residing in less disadvantaged areas (Quintile 4: IRR 1.06, 95% CI 1.02–1.10, p = 0.004). In contrast, opposite associations were observed for the one-quarter-lagged housing debt to income ratio for people residing in more disadvantaged areas (Quintile 2: IRR 0.97, 95% CI 0.93–1.00, p = 0.047) and the one-month-lagged cash rate for people residing in moderately disadvantaged areas (Quintile 3: IRR 0.88, 95% CI 0.78–0.99, p = 0.030).

**Interpretation:**

Our study provides first evidence that the association between macroeconomic changes and suicide varied based on socioeconomic disadvantage. This underscores the need for policies to consider the differential effect to alleviate financial strain to prevent suicide.

**Funding:**

The Australian Government Department of Health, Disability and Ageing.

**Research in context**

**Evidence before this study**

We searched Scopus, Medline, PsycINFO, and Global Health for original articles published from database inception to 29th May 2026, using the search terms “suicid∗” AND (“macroeconomic” OR “economic” OR “financial” OR “inflation” OR “cash rate” OR “interest rate” OR “funds rate” OR “base rate” OR “bank rate” OR “policy rate” OR “overnight rate” OR “operations rate” OR “prime rate” OR “repo rate” OR “savings” OR “mortgage debt” OR “housing debt”). This search identified one American study that showed a stronger adverse effect of economic downturn on suicide for people with fewer than 12 years of education than those with more education years, and one Australian study that showed a more pronounced increase in suicide for economically inactive or unemployed persons than for employed persons during the Global Financial Crisis. Most studies have examined the association between macroeconomic factors (e.g., inflation) and suicide by sex and/or age groups. None of the studies examined these associations across the socioeconomic spectrum.

**Added value of this study**

This policy-driven study presents first evidence of socioeconomic disparities in the association between four macroeconomic changes and suicide. A monthly increase in annual inflation was associated with an increase in suicide rate in the following month for the general population, and for people residing in the most and the least socioeconomic disadvantaged areas. Such increase was also observed in the least disadvantaged group after three months. A quarterly increase in household saving to income ratio was associated with a small increase in suicide rate one quarter later among people residing in the least disadvantaged areas (rising savings may mean lower population-level spending which may adversely impact the businesses/investments of some of these individuals, thereby increasing their financial strain). A quarterly increase in owner-occupier housing debt to income ratio was associated with an increase in suicide rate in the same quarter for individuals residing in the less disadvantaged areas (in which there was a relatively greater proportion of households with large home loans), but a decrease in suicide rate in the following quarter for people residing in more disadvantaged areas (in which there was a relatively less households hold home loans or a greater proportion of households with small home loans). A monthly increase in the cash rate (which tends to signal economic stability and the easing of an economic crisis) was associated with a decrease in suicide rate one month later for people from the moderately disadvantaged areas, but not for other groups.

**Implications of all the available evidence**

Our findings underscore the importance of recognising socioeconomic disparities in the association between macroeconomic changes and suicide when designing policies in response to the current cost-of-living crisis and future economic downturns. These responses include, but are not limited to, timely support for individuals who are struggling with home loan repayments, government financial assistance for necessities, financial counselling and financial literacy programs to cope with financial hardship, and a whole-of-government approach to suicide prevention. Evaluating the effectiveness of these interventions on reducing suicide related to financial stress is needed to identify effective strategies.

## Introduction

The current cost-of-living crisis, which began in late 2021, is adversely affecting every country including Australia.^1^^,^^2^ It was driven by the COVID-19 pandemic which disrupted the global supply chains leading to substantial rises in prices of goods and services (a high level of inflation), and exacerbated by the 2022 invasion of Ukraine, the slowdown of the Chinese economy, climate change, and energy crisis.^1^ In Australia, annual inflation rose by 7.8% in December 2022. Specifically, the annual increase was 9.2% for food prices, 11.7% for electricity, 9.5% for goods, 5.5% for services, and 17.8% for new dwellings.^3^ The current crisis emerged as there was also a prolonged wage stagnation, resulting in the cost of everyday living essentials outpacing the growth of the average household income.^1^ During this time, the benchmark interest rate (e.g., the cash rate in Australia) was sharply increased to reduce inflation, which exacerbated the financial strain faced by individuals with mortgages.^1^^,^^2^ This environment caused extreme financial hardship for many people, particularly those who were already struggling to get by, which raised concern about its impact on suicide.^4^

There is evidence that previous economic downturns and inflation were associated with increases in suicides.^5^^,^^6^ A recent review concluded that economic recession was associated with a heightened risk of suicidal behaviour at both the population and individual levels.^7^ A global scoping review identified seven studies that showed a positive relationship between inflation and suicidal behaviour.^6^ A review of the possible mechanisms and factors for economic recession related suicidal behaviour found that unemployment, job insecurity, financial hardship, bankruptcy, and home repossession resulting from a recession were the major drivers of suicidal behaviour.^8^

Monetary policies also play a role in influencing suicide. Contractionary measures to control inflation can reduce aggregate demand and increase unemployment rates.^9^ These conditions could in turn elevate the risk of suicide.^5^ Increasing the benchmark interest rate is a common monetary policy tool to dampen inflation.^1^ However, this exaggerates financial stress for mortgage holders. A systematic review showed that individuals with unmet loan payments experienced suicidal ideation more often than those without this problem.^10^

While certain socioeconomic groups might be more susceptible to the effect of macroeconomic changes occurring during an economic downturn (e.g., the current cost-of-living crisis) on suicide, evidence on this remains limited. We identified one study that showed the effect of economic downturn on suicide was stronger for individuals with fewer than 12 years of education than those with higher levels of education.^11^ Another study showed that the rise in suicide among economically inactive or unemployed persons was greater than that among employed persons during the Global Financial Crisis.^12^ Addressing this knowledge gap is essential for policymaking given that protective measures, such as social welfare spending^13^ and conditional cash transfers,^14^ that are used to mitigate the impacts of adverse economic conditions on suicide are often targeted according to individual and household socioeconomic circumstances.

Our study aimed to examine the association between changes in four macroeconomic indicators and suicide in Australia across the socioeconomic spectrum. These indicators included annual inflation and the cash rate set by the Reserve Bank of Australia (RBA), which sharply increased during the current cost-of-living crisis. Our study also included indicators closely related to annual inflation and the cash rate ― household saving to income ratio and owner-occupier housing debt to income ratio, which are more direct indicators of financial hardship.

## Methods

In this study, which was conducted using Australian data, we employed an ecological (population-level) time series design. Our study followed the Guidelines for Accurate and Transparent Health Estimates Reporting^15^ (Appendix A).

### Suicide counts

We requested monthly counts of suicides that occurred between January 2006 and October 2023 in Australia for all persons and by five quintile groups of Index of Relative Socio-Economic Disadvantage (IRSD) from the Australian Bureau Statistics (ABS, see Appendix B for the details of ABS suicide data). According to the ABS, an IRSD quintile was assigned to each suicide based on the deceased's area of usual residence at Statistical Area Level 2 (SA2, prior to 2011) or Statistical Area Level 1 (SA1, 2011 or after). The IRSD includes only measures of relative disadvantage and provides a sum score of the economic and social conditions of people and households within an area (see Appendix C for more information). Quintile 1 (low sum score) indicates relatively greater disadvantage and Quintile 5 (high sum score) indicates a relative lack of disadvantage. To ensure the consistency in data quality, we selected year 2006 as the starting point because this year marks the point at which ABS mortality data began undergoing a three-stage revision process to improve data quality and timeliness. We did not include the data from November 2023 onwards because they were incomplete.

### Population estimates

We manually retrieved SA2-level annual estimated resident population data from 2006 to 2023, along with SA2-level IRSD (based on 2021 Census data), from publicly available ABS sources. These data were merged to obtain annual population number by IRSD quintile group, which were subsequently applied to each corresponding year to calculate the monthly/quarterly suicide rate for each group. Around 4% (101/2454) of SA2 areas are not assigned a decile score, either due to small populations or poor data quality.^16^ These SA2 areas were excluded from our analyses by quintile group but not for all persons.

### Exposure variables

We included four continuous macroeconomic indicators: annual inflation (Consumer Price Index-based, measured by percentage change from the corresponding month of previous year), the cash rate, household saving to income ratio, and owner-occupier housing debt to income ratio (see Appendix D for the details of these variables). We used monthly data for the former two indicators and quarterly data for the last two indicators, reflecting their respective data availability.

### Primary analysis

We first set our data as a monthly time series and tested the trend in suicide rates for all groups over time using linear regression models. Suicide rate was the outcome variable, and time was the exposure variable. We also conducted joinpoint regression analysis to identify change points in monthly suicide rates for all groups (see Appendix K for analysis details).

Subsequently, we aggregated suicide data at the monthly level to the quarterly level for the analysis for household saving to income ratio and owner-occupier housing debt to income ratio. This means our analysis was conducted at the quarterly level for these exposures and at the monthly level for annual inflation and cash rate. We then ran Augmented-Dickey-Fuller tests to assess whether outcome and exposure variables had a unit root (non-stationarity). The tests showed that suicide counts for all persons and each quintile group were stationary, but all macroeconomic indicators were non-stationary. We differenced the non-stationary variables to convert them into stationary variables. As a result, we used month-over-month/quarter-over-quarter changes in macroeconomic indicators in our analysis, meaning the exposures represent monthly/quarterly differences. All macroeconomic indicators were lagged by one month/quarter (e.g., monthly change in annual inflation occurred in the previous month, quarterly change in household saving to income ratio in the previous quarter).

We used generalised linear model fitted with Poisson regression^17^ to examine the association between each macroeconomic change and suicide for all persons and by five quintile groups (24 models). We tested for overdispersion of suicide counts for each group using Pearson's chi-square test. Our tests showed that there was evidence of overdispersion for all groups, except the Quintile 4 group. For model consistency across groups, we used Poisson regression (rather than negative binomial regression) and set the scale parameter to the Pearson chi-square statistic divided by the residual degrees of freedom in the model to allow for overdispersion of suicide counts for the groups showing evidence of overdispersion. The log term of the population number for each group in the corresponding year was included as an offset to model the monthly/quarterly suicide rate.

We then determined the appropriate model for all persons and each quintile group (see Appendix E for modelling strategy, Appendix F for model results, and Appendix G for model equations). These models were adjusted for non-linear time trend, seasonality/quarter (if model improved with this variable), and lagged suicide terms (if needed to account for temporal autocorrelation). As unemployment rate has been shown to be associated with suicide^18^ and included macroeconomic indicators,^19^ and may confound the associations between included macroeconomic changes and suicide. All models were thus also adjusted for the confounding effect of unemployment rate obtained from the ABS. The month-over-month/quarter-over-quarter change in unemployment rate was used as unemployment rate was non-stationary. Same as our exposures, this variable was lagged by one month/quarter.

The coefficients in all models were exponentiated into incidence rate ratios (IRRs) and 95% Confidence Intervals (95% CIs). Although statistical significance was defined as p < 0.05, interpretation should consider the 95% CI. All analyses were conducted using Stata/SE 15.1.

### Additional analysis

We performed three additional analyses. Since monthly changes of 1% in annual inflation (only 5 of 214 time points showed this level of change) and cash rate (3 time points showed 1% change) are uncommon in real world settings, we categorised them into groups that better reflect real-world variation. We repeated the primary analysis using these categorical variables. Due to the relatively small sample size (71 time points) for quarterly changes in household saving and housing debt, we did not perform this analysis for these exposures. We also examined the association between macroeconomic changes and suicide, stratified by sex and age group. Sex of suicidal deceased was assigned based on external and internal examination of body characteristics.

### Sensitivity analysis

We performed 14 sensitivity analyses to assess the robustness of our main findings (see Appendix M for the details of these analyses and their results; e.g., analyses without adjusting for unemployment rate).

Ethics approval for this study was obtained from the Human Research Ethics Committee of the University of Melbourne (2025-32385-65821-3). Informed consent was not required for the study because we used aggregate level monthly suicide data provided by the ABS. Data on race or ethnicity were not collected by the data provider (ABS).

### Roles of the funding source

The funders had no role in the design of the study; in the collection, analyses, or interpretation of data; in the writing of the manuscript, or in the decision to publish the results.

## Results

### Trends in macroeconomic indicators and suicide rate

**Fig. 1:**
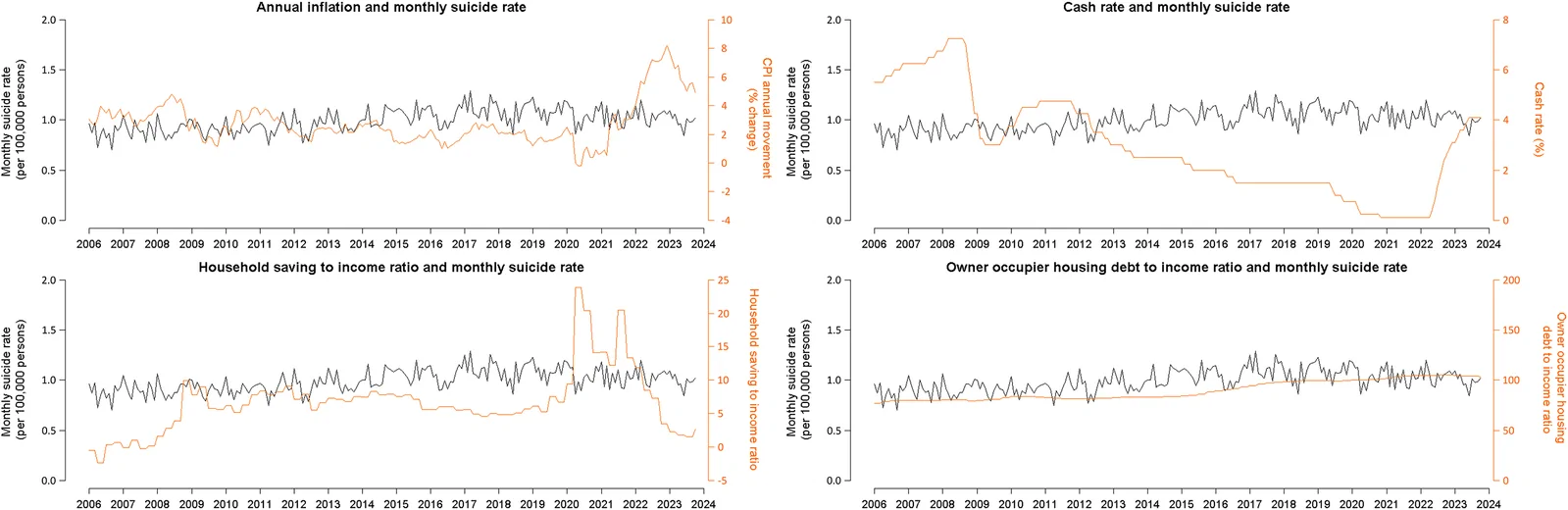
**Monthly/quarterly macroeconomic indicators and monthly suicide rates from January 2006 to October 2023**. Annual inflation and cash rate data are monthly, while household saving to income ratio and owner occupier housing debt to income ratio data are quarterly.

**Fig. 2:**
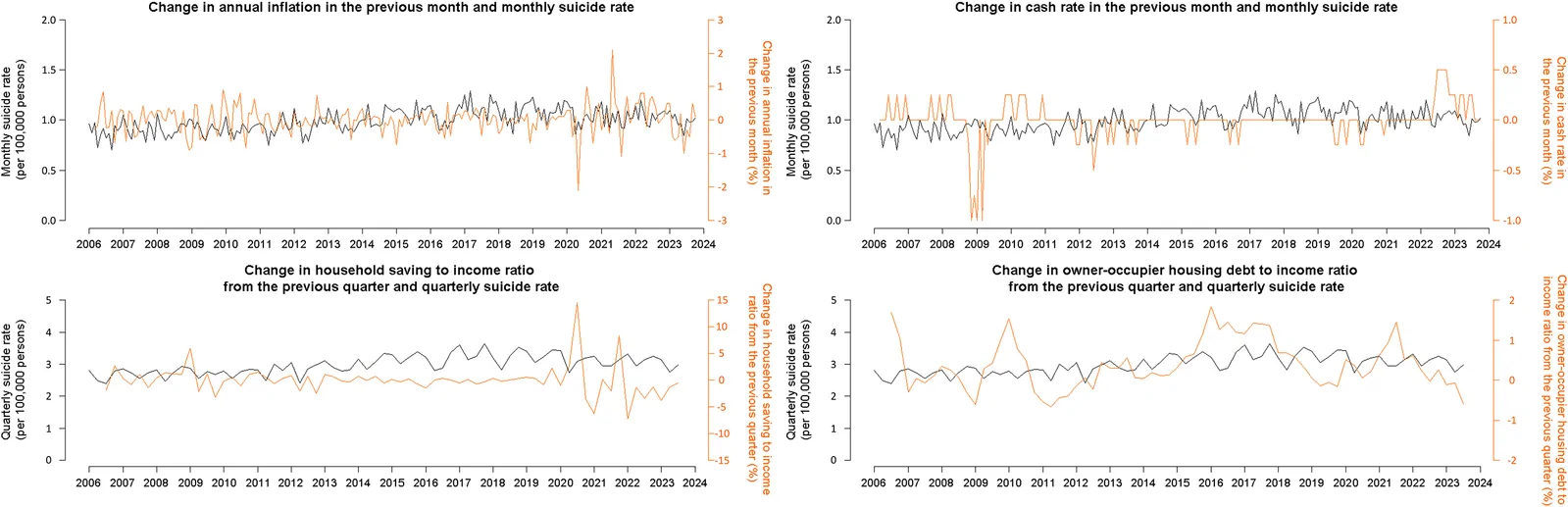
**Monthly/quarterly changes in macroeconomic indicators occurred in the previous month/quarter and monthly/quarterly suicide rates from January 2006 to October 2023**.

The cost-of-living crisis, which began in late 2021, saw a record-breaking increase in annual inflation followed by a decline, a sharp rise in the cash rate since May 2022, a substantial decline in the household saving to income ratio from a peak (23.9%) in the second quarter of 2020 to 1.5% in the third quarter of 2023, and a nearly flat trend (remaining at its highest level throughout the study period) in the owner-occupier housing debt to income ratio (Fig. 1). Fig. 2 shows the monthly/quarterly changes in macroeconomic indicators in the previous month/quarter. There was a sharp drop in annual inflation in April 2020 and a sharp increase in April 2021. The cash rate rose multiple times between May 2022 and June 2023. Household savings increased substantially in 2020 quarter two and declined continuously since 2021 quarter four, while housing debt rose on several occasions throughout the study period (e.g., 2021 quarter two during the COVID-19).

**Fig. 3:**
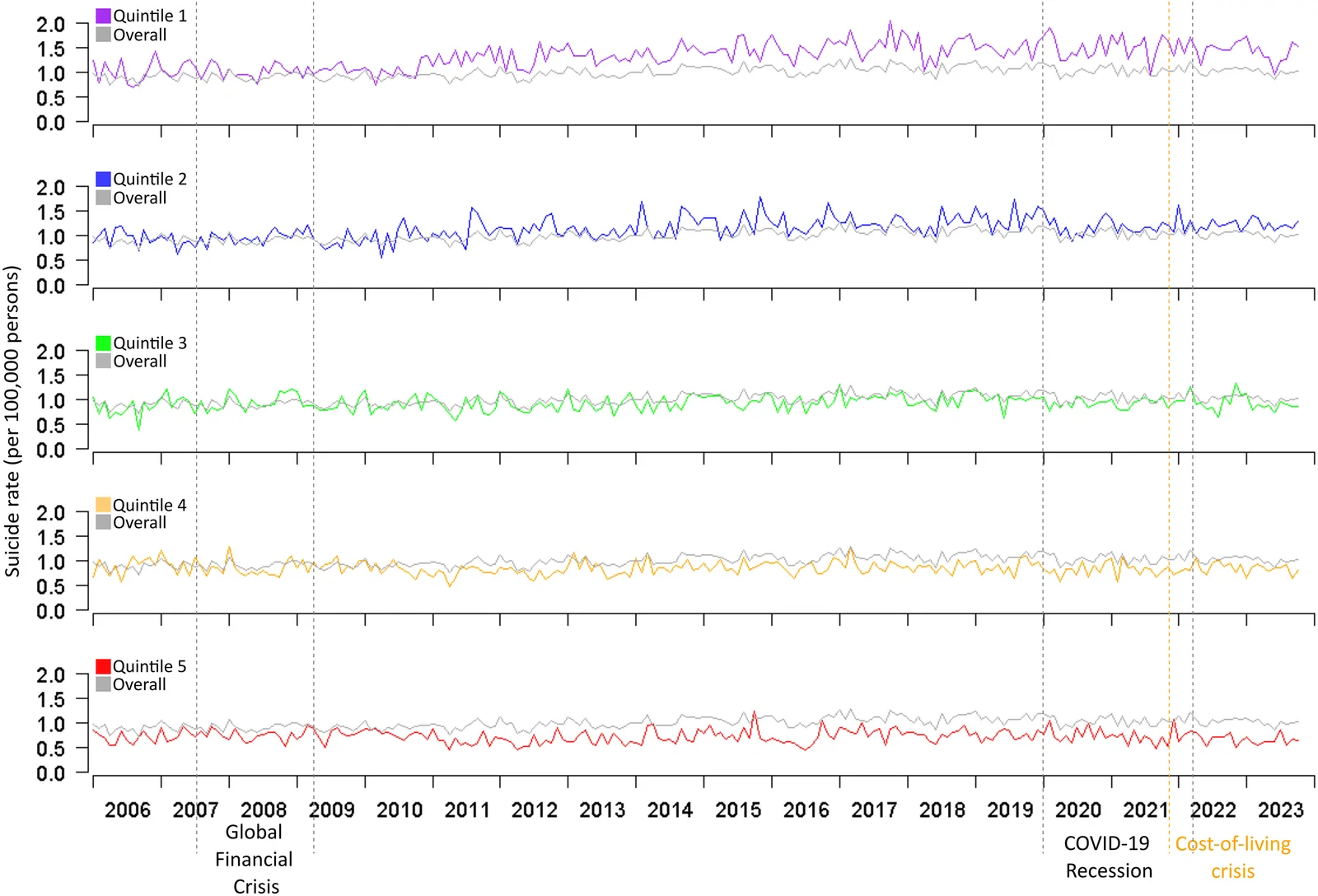
**Monthly suicide rates across groups according to socioeconomic disadvantage (Quintiles 1–5) from January 2006 to October 2023**.

On average, there were 270 suicides per month during the cost-of-living crisis period and 231 suicides per month during the pre-crisis period (Australia's population was approximately 26 million in 2023, Appendix J). The monthly suicide rate for all persons fluctuated between 0.7 and 1.3 per 100,000 persons with a general upward trend (b = 0.0010, p < 0.001; Fig. 1). The average monthly suicide rate was highest for the IRSD Quintile 1 group and consecutively decreased to being the lowest for the IRSD Quintile 5 group (Quintile 1: 1.33 per 100,000 persons; Quintile 2: 1.14 per 100,000 persons; Quintile 3: 0.93 per 100,000 persons; Quintile 4: 0.84 per 100,000 persons; Quintile 5: 0.72 per 100,000 persons; Fig. 3 and Appendix N). IRSD Quintile groups 1–3 showed a general upward trend in their monthly suicide rates (Quintile 1: b = 0.0028, p < 0.001; Quintile 2: b = 0.0018, p < 0.001; Quintile 3: b = 0.0004, p = 0.020), while quintile groups 4 and 5 showed a general flat trend in their monthly suicide rates (Quintile 4: b < −0.0001, p = 0.751; Quintile 5: b < −0.0001, p = 0.866). The joinpoint models did not identify any change points since the cost-of-living crisis for any group (Appendix K), may be because we did not have ‘final’ suicide data for the recent years.

### Macroeconomic changes and suicide rate by socioeconomic group

Table 1 shows that every 1% monthly increase in annual inflation was associated with a 4% increase in suicide rate one month later for the general population (Incidence Rate Ratio (IRR) 1.04, 95% Confidence Interval (CI) 1.01‒1.06, p = 0.011). This positive association was especially robust for people residing in the most and least disadvantaged areas (Quintile 1: IRR 1.07, 95% CI 1.03–1.12, p = 0.001; Quintile 5: IRR 1.07, 95% CI 1.01‒1.14, p = 0.026).

**Table 1:** Association between changes in macroeconomic indicators (lagged by one month/quarter, continuous variable) and monthly/quarterly suicide rate for all persons and across IRSD quintile groups.

	All persons	Quintile 1 (most disadvantage)	Quintile 2	Quintile 3	Quintile 4	Quintile 5 (least disadvantage)
	IRR (95% CI)					
Monthly change in annual inflation in the previous month	1.04 (1.01‒1.06, p = 0.011)^a^	1.07 (1.03–1.12, p = 0.001)^a^	0.98 (0.93–1.03, p = 0.360)	1.00 (0.95–1.06, p = 0.885)	0.99 (0.94–1.04, p = 0.739)	1.07 (1.01‒1.14, p = 0.026)^a^
Monthly change in cash rate in the previous month	1.01 (0.94–1.07, p = 0.857)	1.09 (0.97–1.22, p = 0.156)	0.92 (0.81–1.04, p = 0.184)	0.88 (0.78–0.99, p = 0.030)^a^	1.05 (0.93–1.19, p = 0.412)	1.04 (0.89–1.20, p = 0.637)
Quarterly change in household saving to income ratio in the previous quarter	1.00 (0.99–1.00, p = 0.530)	1.00 (0.99–1.00, p = 0.411)	1.00 (0.99–1.00, p = 0.177)	1.00 (0.99–1.01, p = 0.498)	1.00 (0.99–1.01, p = 0.603)	1.01 (1.00–1.02, p = 0.019)^a^
Quarterly change in owner-occupier housing debt to income ratio in the previous quarter	0.99 (0.97–1.01, p = 0.479)	0.97 (0.94–1.01, p = 0.155)	0.97 (0.93–1.00, p = 0.047)^a^	1.02 (0.98–1.07, p = 0.271)	1.04 (1.00–1.08, p = 0.054)^b^	1.02 (0.97–1.07, p = 0.352)

a Significant result (p < 0.05).

b Marginal significant: “All models were adjusted for non-linear time trend, seasonality (if model improved with this variable), monthly change in unemployment rate (lagged by one month), and lagged suicide terms (if needed). Analyses for annual inflation and cash rate were performed at the monthly level and analyses for household saving to income ratio and housing debt to income ratio were performed at the quarterly level.”

The monthly change in the cash rate (lagged by one month) was negatively associated with suicide rate for people living in the middle-level disadvantaged areas. Specifically, a 1% monthly increase in the cash rate was associated with a 12% decrease in suicide rate in the following month (Quintile 3: IRR 0.88, 95% CI 0.78–0.99, p = 0.030).

The quarterly change in the household saving to income ratio (lagged by one quarter) was positively associated with the quarterly suicide rate for people residing in the least disadvantaged areas. In that, for every 1% quarterly increase in housing saving to income ratio, there was an approximately 1% increase in suicide rate one quarter later among people residing in Quintile 5 areas (Quintile 5: IRR 1.01, 95% CI 1.00–1.02, p = 0.019).

The quarterly change in owner-occupier housing debt to income ratio (lagged by one quarter) was negatively associated with the quarterly suicide rate for people residing in more disadvantaged areas. Among this population, a 1% quarterly increase in this indicator was associated with a 3% decrease in suicide rate one quarter later (Quintile 2: IRR 0.97, 95% CI 0.93–1.00, p = 0.047). It is worth noting that a marginal association was observed for people residing in less disadvantaged areas, in which a 1% quarterly increase in owner-occupier housing debt to income ratio was associated with a 4% increase in suicide rate one quarter later (Quintile 4: IRR 1.04, 95% CI 1.00–1.08, p = 0.054).

### Results from the additional analyses

Appendix L shows the tables for our additional analyses. In the analysis using categorical variable of annual inflation, the monthly suicide rate was greater for all people and for people residing in the most and least disadvantaged areas when there was at least 0.2% monthly increase in annual inflation in the previous month, compared to ≤0 monthly change (All people: IRR 1.03, 95% CI 1.00–1.06, p = 0.037; Quintile 1: IRR 1.05, 95% CI 1.00–1.10, p = 0.038; Quintile 5: IRR 1.09, 95% CI 1.02–1.15, p = 0.006). When the cash rate decreased from the previous month (lagged by one month), the monthly suicide rate was lower for people residing in the most disadvantaged areas compared to when there was no change in the cash rate (Quintile 1: IRR 0.94, 95% CI 0.88–1.00, p = 0.037). However, this finding must be interpreted with caution as the number of time points for the category of a decrease is small.

In the models stratified by sex, a 1% monthly increase in annual inflation was associated with a 4% increase in male suicide rates one month later (IRR 1.04, 95% CI 1.00–1.07, p = 0.025). In the models stratified by age group, a 1% monthly increase in annual inflation was associated with a 9% increase in suicide rates one month later among people aged 70 years and older (IRR 1.09, 95% CI 1.02–1.17, p = 0.011). In contrast, a 1% quarterly increase in household saving to income ratio was associated with a 1% increase in suicide rates one quarter later among people younger than 30 years (IRR 1.01, 95% CI 1.00–1.01, p = 0.014).

### Results from the sensitivity analyses

Appendix M shows the results of our sensitivity analyses and their descriptions. The findings from these sensitivity analyses suggest that the models used in our primary analyses are robust because the estimates remained generally consistent across alternative model specifications (adjusting for different variables, removing unemployment rate as a confounder, and using negative binomial regression). Our findings from the sensitivity analyses showed that the adverse effect of higher housing debt was more immediate for people residing in Quintile 4 areas (IRR 1.06, 95% CI 1.02–1.10, p = 0.004) and the effect of rising inflation persisted three months later for people residing in Quintile 5 areas (IRR 1.07, 95% 1.00–1.13, p = 0.043).

## Discussion

In this Australian study drawing on 214 months/71 quarters (about 18 years) data, we examined the association of changes in annual inflation, the cash rate, household saving to income ratio, and owner-occupier housing debt to income ratio with suicide in the total population and across socioeconomic groups. The effect of these macroeconomic indicators on suicide was not experienced equally and was shaped by socioeconomic disadvantage.

We found that a monthly increase in annual inflation was associated with an increase in suicide rate in the following month for the general population, particularly for individuals residing in the most and least disadvantaged areas. This finding remained when a lower cut-off point of monthly change in annual inflation was used (i.e. ≥ 0.2%). The finding for the overall population is consistent with previous evidence.^6^ For individuals residing in the most disadvantaged areas (which predominantly consist of people with low income and single parent families, spending a larger share of their income on necessities and having little savings buffer), the finding is expected as this population is likely to experience substantial financial strain caused by increase in annual inflation.^20^ The finding for people residing in the least disadvantaged areas is unexpected. For this population, an increase in inflation could potentially induce financial strain by eroding the purchasing power of their cash holdings and fixed-income investments (e.g., bonds), while introducing market volatility resulting in underperforming investments. Individuals who are involved directly in businesses could also have greater difficulty in acquiring necessary funding from financial institutions, which could lead to failed investments (e.g., bankruptcy) and exacerbate their financial strain. These conditions may increase the risk of suicide in some individuals from this group.

Our findings showed that an increase in population-level household saving to income ratio was associated with a small increase in suicide rate among the least disadvantaged group. As a greater proportion of people from this group involved in businesses and investments, rising savings (and lower spending) at the population level may negatively impact their business/investment activity and thereby elevate their financial strain. Although we did not observe negative associations between household saving and suicide for more disadvantaged groups, financial support involving cash transfers to supplement income for families under defined income levels can offer poverty relief, particularly during an economic downturn. A Brazilian study found that beneficiaries of a conditional cash transfer program had a lower suicide risk than non-beneficiaries.^14^ This strategy could be implemented alongside active public initiatives, such as financial counselling and financial literacy programs, in low socioeconomic areas to prevent suicide. Additionally, raising the minimum national wage might also be helpful for this population as it has been shown to be associated with a decline in suicide rates in the United States.^21^^,^^22^

We also found that every 1% quarterly increase in owner-occupier housing debt was associated with a 6% increase in suicide rate during the same quarter for people residing in less disadvantaged areas, indicating a more immediate effect of this macroeconomic change on suicide for this group. Such areas have a relatively greater proportion of households with large mortgage loans.^23^ Increasing housing debt at the population level may mean some individuals within these households are facing a higher level of mortgage stress, thus increasing their risk of suicide. However, the opposite finding was observed for people residing in more disadvantaged areas one quarter later. This may be explained by the relatively low prevalence and magnitude of housing debt in these areas. Fewer households hold housing loans, and those that do generally carry smaller loan repayments. As a result, increases in population-level housing debt may have a limited impact on suicide risk among this group and the negative association may be due to factors such as homeownership and rising property values. Such an association may also reflect the impact of policy interventions during the COVID-19 period, when housing debt increased sharply and people in this group were more likely to qualify for temporary loan repayment deferrals and stimulus payments to help manage their housing debt.^24^^,^^25^

Our results showed that raising the cash rate from the previous month was associated with a reduction in suicide rate one month later among individuals residing in moderately disadvantaged areas (but not among other groups). The RBA raises the cash rate target to help cooldown household spending, which in turn helps bring inflation back down and reduce subsequent cost-of-living pressures. Official announcements of a cash rate increase thus provide a positive signal for economic stability and indicate the easing of an economic crisis. The observed reduction in suicide rate is likely due to the result of improved economic conditions. Alternatively, our results indicate that people in moderately disadvantaged areas were less affected by macroeconomic changes such as increases in annual inflation and housing debt, and may benefit from a relatively more secure financial buffer through interest-bearing investments. However, we note that raising the cash rate often leads to higher mortgage payments, which could heighten suicide risk associated with larger housing debt (as shown in our study). Support for mortgage holders is therefore required during periods of sharp or sustained increases in the cash rate.

Our additional analyses showed that suicide rates in males and older adults (≥70 years) were affected by rising annual inflation. Our finding on males is consistent with that from the previous study, which showed male mortality was positively associated with inflation.^26^ Males are more likely than females to carry financial responsibility,^27^ which may increase their risk of suicide following rising inflation. Among older adults, rising inflation may increase suicide risk by reducing their standard of living. They often spend a higher proportion of income on essentials such as food, healthcare, and utilities, while their pensions, savings, and/or fixed-income investments may not keep up with rising costs. This situation could heighten financial stress and elevate their risk of suicide. Additionally, our findings indicated that an increase in household savings was associated with an increase in suicide rates among people younger than 30 years. A population-level increase in household savings may be driven primarily by older people, meaning that young people are not necessarily saving more and may continue to face financial insecurity and housing unaffordability. Rising savings may also reflect broader economic uncertainty such as during the COVID-19 period, promoting people to save more. Such conditions may be associated with weaker labour market opportunities, job insecurity, and reduced employment prospects, which tend to disproportionally affect young people and may increase their risk of suicide.

This study has several strengths. It is timely as it was conducted during the current cost-of-living crisis and presents first evidence that the association between macroeconomic changes and suicide varied across socioeconomic groups. We used a methodology that yields sensible associations in time series analysis. We also conducted multiple sensitivity analyses to confirm the robustness of our findings.

Our findings should, however, be interpretated with the following limitations. First, we used an ecological design, meaning that the observed associations were at population level and cannot be attributed to individuals. This means although we found that a monthly increase in annual inflation was associated with an increase in suicide for people living in the least disadvantaged areas, we cannot assume that everyone living in the areas was at risk of suicide when annual inflation increased from the previous month. We also recognise that unobserved confounding can never be completely eliminated in research of this nature, although we have implemented several strategies to mitigate this risk. Second, as ‘final’ suicide data were not available for 2021–2023, suicide numbers in these periods might be undercounted. Suicide data are also subject to underreporting for several reasons (e.g., classification of suicide deaths as other causes). Third, although our macroeconomic exposures are commonly used in economic analyses, they may be subject to measurement error. Fourth, we used area-based socioeconomic index to classify the socioeconomic status of the suicide population. This index does not capture individual socioeconomic circumstances. For example, individuals residing in the most disadvantaged areas may not earn a low income. However, this proportion of individuals is small. Fifth, we used annual population numbers to estimate monthly/quarterly suicide rates. Suicide rates might be slightly underestimated. Sixth, our analysis did not consider interventions deployed during the COVID-19 pandemic (e.g., JobKeeper Payment for businesses impacted by the COVID-19 to retain employees, temporary home loan repayment deferrals),^25^ which may have buffered the suicide risk for certain socioeconomic groups during this period. This could affect the observed associations (e.g., housing debt). Seventh, our study was conducted using Australian data. This means our findings may not apply to other countries. Eighth, we recognise that the effects of macroeconomic changes on suicide likely vary across regions, as reflected in the differing distribution of dwellings by IRSD quintile across states and territories (Appendix O), indicating that socioeconomic disadvantage is not uniformly distributed geographically. These effects may also vary according to state- and territory-specific policy responses during periods of economic stress. However, due to the unavailability of region-level data at the time of data collection, we were unable to examine regional variation in our study. Nineth, our analyses did not adjust for multiplicity, and the results should be interpreted accordingly. Finally, the absence of clear associations between macroeconomic changes and suicide among females may reflect lower statistical power, as female suicides accounted only for approximately 25% of all suicides.

In conclusion, our findings suggest that specific socioeconomic groups may be more vulnerable to suicide during adverse macroeconomic changes (i.e. increases in annual inflation, owner-occupier housing debt to income ratio, and household saving to income ratio). These associations suggest that multisectoral policies targeting socioeconomic disparities to mitigate financial stress are important for preventing suicide.

## Contributors

PCFL: data curation, formal analysis, visualisation, writing–original draft, writing–review & editing; LR: project administration, writing–review & editing; PC: formal analysis, writing–review & editing; MD: formal analysis, writing–review & editing; ST: formal analysis, writing–review & editing; SS: writing–review & editing; JP: writing–review & editing; GA: writing–review & editing; VA: writing–review & editing; KH: writing–review & editing; MS: writing–review & editing; EG: writing–review & editing; LST: conceptualisation, methodology, validation, formal analysis, funding acquisition, writing–review & editing. LST verified the data, PCFL and LST had access to raw data, and LST had final responsibility for the decision to submit for publication. MD, JP, GA, and KH are full professors.

## Data sharing statement

Monthly/quarterly data on household saving to income ratio, the cash rate, owner-occupier housing debt to income ratio, unemployment rate is available in Appendix I. Monthly data on suicide and annual inflation were requested from the relevant data custodians, and only LST and PL were granted the access to the data for this study.

## Declaration of interests

The authors declare no conflict of interest.
