# Supplementary material for: Socioeconomic disparities in the association between macroeconomic changes and suicide: a population-level time series study in Australia
Source: Lancet Reg Health West Pac. 2026 Jul 18;73:101927. doi: 10.1016/j.lanwpc.2026.101927 (PMC13540671; doi:10.1016/j.lanwpc.2026.101927)
Supplement: Supplementary file 1 [file mmc1.docx]

**Supplementary appendix**

This appendix formed part of the original submission.

Supplement to: Socioeconomic disparities in the association between macroeconomic changes and suicide: a population-level time series study in Australia

Supplementary materials

[Appendix A. Compliance with the Guidelines for Accurate and Transparent Health Estimates Reporting (GATHER)	3](#_Toc226975773)

[Appendix B. Suicide data from the Australian Bureau of Statistics	4](#_Toc226975774)

[Appendix C. Details of Index of Relative Socio-Economic Disadvantage (IRSD)	5](#_Toc226975775)

[Appendix D: Details of macroeconomic exposures	7](#_Toc226975776)

[Appendix E. Data modelling strategy	8](#_Toc226975777)

[Appendix F. Model results	9](#_Toc226975778)

[Appendix G. Model equations	10](#_Toc226975779)

[Appendix H. Stata code used to generate main results	11](#_Toc226975780)

[Appendix I. Monthly/quarterly publicly available data on macroeconomic indicators	16](#_Toc226975781)

[Appendix J. Suicide count and population number by year	22](#_Toc226975782)

[Appendix K. Joinpoint regression analyses and results	23](#_Toc226975783)

[Appendix L. Additional analyses and results	24](#_Toc226975784)

[Appendix M. Sensitivity analyses and results	27](#_Toc226975785)

[Appendix N. Supplementary figures	40](#_Toc226975786)

[Appendix O. Proportion of dwellings by IRSD group across region	45](#_Toc226975787)

[Appendix P. Data handling procedures	46](#_Toc226975788)

# Appendix A. Compliance with the Guidelines for Accurate and Transparent Health Estimates Reporting (GATHER)

***Table S1.* GATHER**

| **Item number** | **Checklist item** | **Page number** |
| --- | --- | --- |
| **Objective and funding** | |  |
| 1 | Define the indicator(s), populations (including age, sex, and geographic entities), and time period(s) for which estimates were made. | Pages 7-8 |
| 2 | List the funding sources for the work. | Abstract; pages 19-20 |
| **Data inputs** | |  |
| *For all data inputs from multiple sources that are synthesised as part of the study:* | |  |
| 3 | Describe how the data were identified and how the data were accessed. | Pages 7‒8; Appendix D |
| 4 | Specify the inclusion and exclusion criteria. Identify all ad-hoc exclusions. | Pages 7‒8 |
| 5 | Provide information about all included data sources and their main characteristics. For each data source used, report reference information or contact name/institution, population represented, data collection method, year(s) of data collection, sex and age range, diagnostic criteria or measurement method, and sample size, as relevant. | Pages 7‒8; Appendix D |
| 6 | Identify and describe any categories of input data that have potentially important biases (e.g., based on characteristics listed in item 5). | Pages 8-10 |
| *For data inputs that contribute to the analysis but were not synthesised as part of the study:* | |  |
| 7 | Describe and give sources for any other data inputs. | Appendix D |
| *For all data inputs:* | |  |
| 8 | Provide all data inputs in a file format from which data can be efficiently extracted (eg, a spreadsheet rather than a PDF), including all relevant meta-data listed in item 5. For any data inputs that cannot be shared because of ethical or legal reasons, such as third-party ownership, provide a contact name or the name of the institution that retains the right to the data. | Appendix D |
| **Data analysis** | |  |
| 9 | Provide a conceptual overview of the data analysis method. A diagram may be helpful. | Pages 8‒10; Appendix E |
| 10 | Provide a detailed description of all steps of the analysis, including mathematical formulae. This description should cover, as relevant, data cleaning, data pre-processing, data adjustments and weighting of data sources, and mathematical or statistical model(s). | Pages 8‒10; Appendices E, F, and G |
| 11 | Describe how candidate models were evaluated and how the final model(s) were selected. | Pages 8‒10; Appendices E, F, and G |
| 12 | Provide the results of an evaluation of model performance, if done, as well as the results of any relevant sensitivity analysis. | Pages 8‒10, Appendices F, L, M |
| 13 | Describe methods of calculating uncertainty of the estimates. State which sources of uncertainty were, and were not, accounted for in the uncertainty analysis. | Page 10 |
| 14 | State how analytical or statistical source code used to generate estimates can be accessed. | Appendix H |
| **Results and discussion** | |  |
| 15 | Provide published estimates in a file format from which data can be efficiently extracted. | Appendix I |
| 16 | Report a quantitative measure of the uncertainty of the estimates (eg, uncertainty intervals). | Pages 11‒14 |
| 17 | Interpret results in light of existing evidence. If updating a previous set of estimates, describe the reasons for changes in estimates. | Pages 14‒17 |
| 18 | Discuss limitations of the estimates. Include a discussion of any modelling assumptions or data limitations that affect interpretation of the estimates. | Page 17 |

# Appendix B. Suicide data from the Australian Bureau of Statistics

Monthly intentional self-harm (suicide) deaths by IRSD quintiles from 2006 to 2023 were provided by the Australian Bureau of Statistics (ABS). ABS is the national statistical agency in Australia. It provides reliable official statistics on key economic, social, population, and environmental matters. ABS’s suicide data used in this study were compiled by the year in which the suicide death occurred, and contained final data for 2006 ̶ 2020, revised data for 2021, preliminary revised data for 2022, and preliminary data for 2023. The 10th revision of the International Classification of Diseases (ICD-10) codes: X60‒X84 and Y87.0 are used by the ABS to classify intentional self-harm.^1^

All causes of death data from 2006 onward are subject to a revisions process.^1^ Since 2006, when the ABS first releases the causes of death data for a reference year, the data is ‘preliminary’. This is because some deaths (e.g., died by suicide) take time to be fully investigated by a coroner, which can lead to the delay in assigning a cause of death code during the ABS coding process. As further updates on a coronial case are provided to the ABS, the death is re-examined by the mortality coding team and the death is assigned an updated ICD-10 codes if necessary. This revisions process is conducted three times by the ABS – a preliminary revision, a secondary revision and a final revision. Once data for a reference year are 'final', they are no longer revised. The number of suicide deaths typically increase over the revisions period. Suicide counts were estimated to increase by an average of 2·2% from preliminary to final estimates between 2014 and 2020.^2^ There is also jurisdiction variation in the impact of revisions on suicide counts.^2^ For these reasons, results from the time series analysis for data that is not final should be interpreted with caution.

For the suicide data provided by the ABS that used in our analysis, data cells with small values are randomly assigned to protect the confidentiality of individuals. As a result, some totals may not equal the sum of their components. Cells with a zero value are not affected by confidentialisation.

Suicide data are subject to underreporting for several reasons, including misclassification of suicide deaths as other causes, insufficient information to determine suicidal intent, inconsistent coronial processes for determining intent, and social factors such as stigma associated with suicide.^3^

^1^Australian Bureau of Statistics. Causes of Death, Australia methodology; 2023.

^2^Australian Bureau of Statistics. Causes of Death; 2023.

^3^De Leo D, Dudley MJ, Aebersold CJ, Mendoza J, Barnes MA, Harrison JE, et al. Achieving standardised reporting of suicide in Australia: rationale and program for change. *Med J Aust* 2010; **192**: 452–456.

# Appendix C. Details of Index of Relative Socio-Economic Disadvantage (IRSD)

In Australia, Socio-Economic Indexes for Areas (SEIFA) is a commonly used measure that summarise the socio-economic characteristics of an area. SEIFA was developed by the Australian Bureau of Statistics (ABS) and have been updated every five years based on information from the five-yearly Census.^4^ The latest SEIFA was constructed using the 2021 Census data. SEIFA combines Census data on income, education, employment, occupation, housing and family structure. There are four SEIFA indexes:

- The Index of Relative Socio-economic Advantage and Disadvantage (IRSAD), which focuses on both advantage and disadvantage of the socio-economic conditions in an area
- The Index of Relative Socio-economic Disadvantage (IRSD), which focuses on relative socio-economic disadvantage in an area
- The Index of Education and Occupation (IEO), which focuses on relative education and occupation advantage and disadvantage in an area
- The Index of Economic Resources (IER), which focuses on economic advantage and disadvantage in an area

SEIFA 2021 is available at the Statistical Area Level 1 (SA1), Statistical Area Level 2 (SA2), Local Government Areas (LGA), Suburbs and Localities (SAL), and Postal Areas (POA). SEIFA quintiles are based on aggregate quintiles of SA2 (pre-2011) or SA1 (2011 or later) areas where the SA1 previous Census SEIFA score is within the defined national quintile. As such, individual SA1s/SA2s can be in different quintiles after each Census.

Our study used The Index of Relative Socio-economic Disadvantage (IRSD), which was constructed using 16 variables (Table S2). It can be used to understand an area's relative disadvantage and lack of disadvantage. A low score of IRSD indicates relatively greater disadvantage in general, whereas a high score indicates a relative lack of disadvantage.^5^ For example, an area with a low score contains many households with low income, many people with no qualifications, or many people in low skill occupations. An area with a high score consists of few households with low incomes, few people with no qualifications, and few people in low skilled occupations.

^4^Australian Bureau of Statistics. Socio-Economic Indexes for Areas (SEIFA): Technical Paper; 2021.

^5^Australian Bureau of Statistics. 22033.0.55.001 Census of Population and Housing: Socio-Economic Indexes for Areas (SEIFA), Australia; 2011.

***Table S2*. The 16 variables included in the IRSD and their loadings^4^**

| **No.** | **Variable** | **loading** |
| --- | --- | --- |
| 1 | % of people who do not speak English well | -0·34 |
| 2 | % of people aged 15 years and over who have no educational attainment | -0·44 |
| 3 | % of employed people classified as low skill Community and Personal Service workers | -0·50 |
| 4 | % of employed people classified as Machinery Operators and Drivers | -0·52 |
| 5 | % of occupied private dwellings requiring one or more extra bedrooms | -0·52 |
| 6 | % of people aged 15 years and over who are separated or divorced | -0·54 |
| 7 | % of occupied private dwellings with no cars | -0·56 |
| 8 | % of people under the age of 70 who have a long-term health condition or disability and need assistance with core activities | -0·66 |
| 9 | % of one parent families with dependent offspring only | -0·71 |
| 10 | % of occupied private dwellings paying rent less than $166 per week (excluding $0 per week) | -0·73 |
| 11 | % of people (in the labour force) who are unemployed | -0·74 |
| 12 | % of people aged 15 years and over whose highest level of education is Year 11 or lower | -0·75 |
| 13 | % of employed people classified as Labourers | -0·75 |
| 14 | % of occupied private dwellings with no internet connection | -0·81 |
| 15 | % of families with children under 15 years of age who live with jobless parents | -0·85 |
| 16 | % of people with stated household equivalised income between $1 and $20,799 per year | -0·90 |

The loading for each variable is closely related to the variable weights used in constructing the index.

# Appendix D: Details of macroeconomic exposures

***Table S3*. Details of macroeconomic exposures**

| Variable | Expression | Description | Data frequency and period | Data source |
| --- | --- | --- | --- | --- |
| Exposure variables | | | | |
| Consumer Price Index (CPI)-based annual inflation | Percentage change from corresponding month of previous year (seasonally adjusted) | A metric that measures change in prices of goods and services, used to gauge the bare minimum cost required to maintain a given standard of living. A higher CPI generally indicates that households need to spend more to maintain the same standard of living. | Monthly, 2006‒2023 | Australian Bureau of Statistics, Melbourne Institute of Applied Economic and Social Research |
| Household saving to income ratio | Percentage (current price measure, seasonally adjusted) | A metric that measures a household disposable income that is saved, used to gauge financial hardship. A higher percentage generally indicates that households are more financially affluent. | Quarterly, 2006‒2023  (quarterly data was applied to the household saving to income ratio for each corresponding month) | Australian Bureau of Statistics |
| The Reserve Bank of Australia’s cash rate | Percentage | The interest rate on unsecured overnight loans between banks, set by the Reserve Bank of Australia to influence economic activity and inflation. Setting a higher cash rate generally discourages borrowing and spending (i.e., encourages savings) which can reduce inflation (by lowering demand for goods). | Monthly, 2006‒2023 | Reserve Bank Australia |
| Owner-occupier housing debt to income ratio | Percentage (current price measure, seasonally adjusted) | A metric that is calculated as a ratio of owner-occupier housing debt to annualised household disposable income. A higher percentage generally indicates increasing housing costs for mortgage holders relative to income. | Quarterly, 2006‒2023  (quarterly data was applied to the owner-occupier housing debt to income for each corresponding month) | Australian Bureau of Statistics |
| Confounder | | | | |
| Unemployment rate | Percentage (seasonally adjusted) | The proportion of people in the labour force who are unemployed. | Monthly, 2006‒2023 | Australian Bureau of Statistics |

These macroeconomic exposures are commonly used in economic analyses, although they may be subject to measurement error. Household saving to income ratio and housing debt to income ratio were available only on a quarterly basis. These macroeconomic indicators were weakly correlated (r < 0·38).

# Appendix E. Data modelling strategy

We took the following steps to determine the best-fitting model for all persons and each Quintile group to ensure the observed associations are reliable:

1. We searched for the best-fitting time trend for each group using fractional polynomial transformation to the variable of time and adjusted for the best-fitting time trends in the models.
2. We tested whether adding seasonality in the models significantly improved the model fit using the likelihood ratio test. Seasonality was added to the models if there was evidence of model improvement. Categorical seasonality was generated based on four seasons rather than using Fourier terms (sine and cosine pairs) because the former assumes a fixed season structure (e.g., holidays, seasons) while the latter assumes a smooth cyclical pattern (e.g., temperature). The former assumption was supported by previous studies showing that suicide rates peaked in certain seasons such as spring and autumn.^6,7^
3. We assessed autocorrelation in our time series data using Portmanteau’s test for white noise. If there was evidence of autocorrelation, lagged terms for suicide counts were added into the model until this test showed that there was no evidence of autocorrelation.

^6^To S, Messias E, Burch L, Chibnall J. Seasonal variation in suicide: age group and summer effects in the United States (2015–2020). *BMC Psychiatry* 2024; **24**: 856.

^7^Yu J, Yang D, Kim Y, et al. Seasonality of suicide: a multi-country multi-community observational study. *Epidemiol Psychiatr Sci* 2020; **29**: e163.

# Appendix F. Model results

***Table S4.* Model results for all quintiles and each quintile group derived from our modelling strategy**

| **IRSD** | **Suicide data stationarity** | **Overdispersion** | **Time trend and/or seasonality variables**  **(analysis at the monthly level)** | **Lagged suicide term to control autocorrelation (analysis at the monthly level)** | **Time trend and/or seasonality variables**  **(analysis at the quarterly level)** | **Lagged suicide term to control autocorrelation (analysis at the quarterly level)** |
| --- | --- | --- | --- | --- | --- | --- |
| All persons | Evidence of stationarity | Evidence of overdispersion (monthly and quarterly suicide data) | Non-linear time trend (powers 2 3) and seasonality | 12 | Non-linear time trend (powers 3 3) and seasonality | 0 |
| Quintile 1  (most disadvantaged) | Evidence of stationarity | Evidence of overdispersion (monthly and quarterly suicide data) | Non-linear time trend (powers 2 2) and seasonality | 0 | Non-linear time trend (powers 2 2) and seasonality | 0 |
| Quintile 2 | Evidence of stationarity | Evidence of overdispersion (monthly and quarterly suicide data) | Non-linear time trend (powers 2 2) and seasonality | 3 | Non-linear time trend (powers 2 2) and seasonality | 1 |
| Quintile 3 | Evidence of stationarity | Evidence of overdispersion (monthly and quarterly suicide data) | Non-linear time trend (powers 3 3) and seasonality | 0 | Non-linear time trend (powers -2 -2) and seasonality | 0 |
| Quintile 4 | Evidence of stationarity | No evidence of overdispersion (monthly and quarterly suicide data) | Non-linear time trend (powers -2 -1) | 0 | Non-linear time trend (powers -2 -1) and seasonality | 0 |
| Quintile 5  (least disadvantaged) | Evidence of stationarity | Evidence of overdispersion (monthly and quarterly suicide data) | Non-linear time trend (powers 3 3) | 0 | Non-linear time trend (powers 3 3) and seasonality | 1 |

# Appendix G. Model equations

The model equation for the association between macroeconomic changes and suicide for all persons at the monthly level was^8^:

log(y*_t_*) = *β*_0_ + *β*_1_ dcpi*_t-1_* + *β*_2_ dunemployrate*_t-1_* + *β*_3_ time_1*_t_* + *β*_4_ time_2*_t_* + *γ*_1_ *I* (Fall*_t_*) + *γ_2_* *I* (Winter*_t_*) + *γ_3_* *I* (Spring*_t_*) + $\sum_{k=1}^{12} \delta$*_k_* suicide*_t-k_* + log(pop*_t_*)

The outcome *(y_t_)* is the count of the number of suicides in month *t*. The predictor is (dcpi*_t-1_*) monthly change in annual inflation in month *t-1*. The controlled variables included monthly change in unemployment rate **(**dunemployrate) in month *t-1*, non-linear time trend terms (time_1, time_2_),_ season (three dummy variables for fall, winter, and spring with summer as the reference group). Our model also included an autoregression term (suicide *_t-k_*): suicide counts in the previous *k* months.

For models with cash rate, one-month lag of monthly change in annual inflation was replaced with one-month lag of monthly change in cash rate. See Table S4 for controlled variables for each analysis.

The model equation for the association between macroeconomic changes and suicide for all persons at the quarterly level was^8^:

log(y*_t_*) = *β*_0_ + *β*_1_ dhs*_t-1_* + *β*_2_ dunemployrate*_t-1_* + *β*_3_ time_1*_t_* + *β*_4_ time_2*_t_* + *γ*_1_ *I* (Quarter2*_t_*) + *γ_2_* *I* (Quarter3*_t_*) + *γ_3_* *I* (Quarter4*_t_*) + log(pop*_t_*)

The outcome *(y_t_)* is the count of the number of suicides in quarter *t*. The predictor is (dhs*_t-1_*) quarterly change in household saving to income ratio in quarter *t-1*. The controlled variables included quarterly change in unemployment rate **(**dunemployrate) in quarter *t-1*, non-linear time trend terms (time_1, time_2_),_ quarter (three dummy variables for Quarter 2, Quarter 3, and Quarter 4 with Quarter 1 as the reference group). An autoregression term is not required as there was no evidence of autocorrelation for suicide among all persons.

For models with housing debt, one-quarter lag of quarterly changes in household saving was replaced with one-quarter lag of quarterly change in housing debt.

^8^Davis RA, Fokianos K, Holan SH, et al. Count time series: a methodological review*. J Am Stat Assoc* 2021; 166:1–50.

# Appendix H. Stata code used to generate main results

** Set file path

cd "C:\Macroeconomic and Suicide Study"

************************ For analysis at monthly level ************************

** Use data

use "monthlysuicide_irsd.dta", clear

** Create IRSD variable

gen irsd = cond(seifa == "Quintile 1 (lowest)", 1, ///

cond(seifa == "Quintile 2", 2, ///

cond(seifa == "Quintile 3", 3, ///

cond(seifa == "Quintile 4", 4, ///

cond(seifa == "Quntile 5 (highest)", 5, ///

cond(seifa == "All", 6, . ))))))

label define irsd ///

1 "[1] Q1 (lowest)" ///

2 "[2] Q2" ///

3 "[3] Q3" ///

4 "[4] Q4" ///

5 "[5] Q5 (highest)" ///

6 “[6] all”

label values irsd irsd

label variable irsd "IRSD group"

** Season categorical to control for holidays

gen season = .

replace season = 1 if inlist(month, 12, 1, 2)

replace season = 2 if inlist(month, 3, 4, 5)

replace season = 3 if inlist(month, 6, 7, 8)

replace season = 4 if inlist(month, 9, 10, 11)

label define season 1 "Summer" 2 "Fall" 3 "Winter" 4 "Spring"

label values season season

** log population size

gen log_pop = ln(population)

** Sort month

sort mth

gen time = _n

** Drop last two months

drop if time == 215

drop if time == 216

** Set data as time-series

tsset time

** Trend of suicide rate

Gen suirate = suicide/population*100,000

reg suirate time

** Stationary test

dfuller suicide

dfuller cpi_change

gen dcpi = D.cpi

dfuller dcpi

dfuller cashrate

gen dcashrate = D.cashrate

dfuller dcashrate

dfuller unemployment

gen dunemployrate = D.unemployment

dfuller dunemployrate

** Overdispersion test

poisson suicide, offset(log_pop) vce(robust)

estat gof, pearson

** Finding the best-fitting time trend

fp <time>: glm suicide <time>, family(poisson) link(log) scale(x2) offset(log_pop)

** Compare models with and without seasonality

glm suicide lag1dcpi lag1dunemployrate time_1 time_2 , family(poisson) link(log) scale(x2) offset(log_pop)

est store model1

glm suicide lag1dcpi lag1dunemployrate time_1 time_2 i.season, family(poisson) link(log) scale(x2) offset(log_pop)

est store model2

estimate stats model1 model2

lrtest model1 model2

** Autocorrelation check

glm suicide lag1dcpi lag1dunemployrate time_1 time_2 i.season, family(poisson) link(log) scale(x2) offset(log_pop) eform

predict resid1, response

wntestq resid1

** Gen lag term exposures

gen lag1dcpi = L1.dcpi

gen lag1dcashrate = L1.dcashrate

gen lag1dunemployrate = L1.dunemployrate

** Generalised linear model fitted with Poisson regression

** All persons

glm suicide lag1dcpi lag1dunemployrate time_1 time_2 i.season L(1/12).suicide, family(poisson) link(log) scale(x2) offset(log_pop) eform cformat(%8.2f)

glm suicide lag1dcashrate lag1dunemployrate time_1 time_2 i.season L(1/12).suicide, family(poisson) link(log) scale(x2) offset(log_pop) eform cformat(%8.2f)

** Q1

glm suicide lag1dcpi lag1dunemployrate time_1 time_2 i.season, family(poisson) link(log) scale(x2) offset(log_pop) eform cformat(%8.2f)

glm suicide lag1dcashrate lag1dunemployrate time_1 time_2 i.season, family(poisson) link(log) scale(x2) offset(log_pop) eform cformat(%8.2f)

** Q2

glm suicide lag1dcpi lag1dunemployrate time_1 time_2 i.season L(1/3).suicide, family(poisson) link(log) scale(x2) offset(log_pop) eform cformat(%8.2f)

glm suicide lag1dcashrate lag1dunemployrate time_1 time_2 i.season L(1/3).suicide, family(poisson) link(log) scale(x2) offset(log_pop) eform cformat(%8.2f)

** Q3

glm suicide lag1dcpi lag1dunemployrate time_1 time_2 i.season, family(poisson) link(log) scale(x2) offset(log_pop) eform cformat(%8.2f)

glm suicide lag1dcashrate lag1dunemployrate time_1 time_2 i.season, family(poisson) link(log) scale(x2) offset(log_pop) eform cformat(%8.2f)

** Q4

glm suicide lag1dcpi lag1dunemployrate time_1 time_2, family(poisson) link(log) offset(log_pop) eform cformat(%8.2f)

glm suicide lag1dcashrate lag1dunemployrate time_1 time_2, family(poisson) link(log) offset(log_pop) eform cformat(%8.2f)

** Q5

glm suicide lag1dcpi lag1dunemployrate time_1 time_2, family(poisson) link(log) scale(x2) offset(log_pop) eform cformat(%8.2f)

glm suicide lag1dcashrate lag1dunemployrate time_1 time_2, family(poisson) link(log) scale(x2) offset(log_pop) eform cformat(%8.2f)

************************ For analysis at quarterly level ************************

** Use data

use "qrtlysuicide_irsd.dta", clear

** Season categorical (approximate)

gen season = .

replace season = 1 if quarter==1

replace season = 2 if quarter==4

replace season = 3 if quarter==7

replace season = 4 if quarter==10

label define season 1 "Summer" 2 "Fall" 3 "Winter" 4 "Spring"

label values season season

* Sort quarter

sort qrt

gen time = _n

drop if time == 72

// All persons

glm suicide lag1dhsave lag1dunemployrate time_1 time_2 i.season, family(poisson) link(log) scale(x2) offset(log_pop) eform cformat(%8.2f)

glm suicide lag1dodebt lag1dunemployrate time_1 time_2 i.season, family(poisson) link(log) scale(x2) offset(log_pop) eform cformat(%8.2f)

// Person Q1

glm suicide lag1dhsave lag1dunemployrate time_1 time_2 i.season, family(poisson) link(log) scale(x2) offset(log_pop) eform cformat(%8.2f)

glm suicide lag1dodebt lag1dunemployrate time_1 time_2 i.season, family(poisson) link(log) scale(x2) offset(log_pop) eform cformat(%8.2f)

// Person Q2

glm suicide lag1dhsave lag1dunemployrate time_1 time_2 i.season L.suicide, family(poisson) link(log) scale(x2) offset(log_pop) eform cformat(%8.2f)

glm suicide lag1dodebt lag1dunemployrate time_1 time_2 i.season L.suicide, family(poisson) link(log) scale(x2) offset(log_pop) eform cformat(%8.2f)

// Person Q3

glm suicide lag1dhsave lag1dunemployrate time_1 time_2 i.season, family(poisson) link(log) scale(x2) offset(log_pop) eform cformat(%8.2f)

glm suicide lag1dodebt lag1dunemployrate time_1 time_2 i.season, family(poisson) link(log) scale(x2) offset(log_pop) eform cformat(%8.2f)

// Person Q4

glm suicide lag1dhsave lag1dunemployrate time_1 time_2, family(poisson) link(log) offset(log_pop) eform cformat(%8.2f)

glm suicide lag1dodebt lag1dunemployrate time_1 time_2, family(poisson) link(log) offset(log_pop) eform cformat(%8.2f)

// Person Q5

glm suicide lag1dhsave lag1dunemployrate time_1 time_2 i.season, family(poisson) link(log) scale(x2) offset(log_pop) eform cformat(%8.2f)

glm suicide lag1dodebt lag1dunemployrate time_1 time_2 i.season, family(poisson) link(log) scale(x2) offset(log_pop) eform cformat(%8.2f)

# Appendix I. Monthly/quarterly publicly available data on macroeconomic indicators

***Table S5.* Monthly/quarterly data on macroeconomic factors**

| **Month and year** | **Household saving to income ratio** | **Cash rate** | **Owner occupier housing debt to income ratio** | **Unemployment rate** |
| --- | --- | --- | --- | --- |
| Jan 2006 | -0·50 | 5·50 | 77·16 | 5·16 |
| Feb 2006 | -0·50 | 5·50 | 77·16 | 5·10 |
| Mar 2006 | -0·50 | 5·50 | 77·16 | 4·87 |
| Apr 2006 | -2·40 | 5·50 | 78·85 | 4·96 |
| May 2006 | -2·40 | 5·75 | 78·85 | 4·80 |
| Jun 2006 | -2·40 | 5·75 | 78·85 | 4·81 |
| Jul 2006 | 0·30 | 5·75 | 79·91 | 4·66 |
| Aug 2006 | 0·30 | 6·00 | 79·91 | 4·71 |
| Sep 2006 | 0·30 | 6·00 | 79·91 | 4·67 |
| Oct 2006 | 0·70 | 6·00 | 79·62 | 4·46 |
| Nov 2006 | 0·70 | 6·25 | 79·62 | 4·51 |
| Dec 2006 | 0·70 | 6·25 | 79·62 | 4·57 |
| Jan 2007 | -0·10 | 6·25 | 79·67 | 4·55 |
| Feb 2007 | -0·10 | 6·25 | 79·67 | 4·63 |
| Mar 2007 | -0·10 | 6·25 | 79·67 | 4·46 |
| Apr 2007 | 1·00 | 6·25 | 79·60 | 4·39 |
| May 2007 | 1·00 | 6·25 | 79·60 | 4·26 |
| Jun 2007 | 1·00 | 6·25 | 79·60 | 4·32 |
| Jul 2007 | -0·30 | 6·25 | 79·70 | 4·25 |
| Aug 2007 | -0·30 | 6·50 | 79·70 | 4·30 |
| Sep 2007 | -0·30 | 6·50 | 79·70 | 4·21 |
| Oct 2007 | 0·20 | 6·50 | 80·05 | 4·33 |
| Nov 2007 | 0·20 | 6·75 | 80·05 | 4·43 |
| Dec 2007 | 0·20 | 6·75 | 80·05 | 4·31 |
| Jan 2008 | 1·60 | 6·75 | 80·31 | 4·22 |
| Feb 2008 | 1·60 | 7·00 | 80·31 | 3·98 |
| Mar 2008 | 1·60 | 7·25 | 80·31 | 4·06 |
| Apr 2008 | 2·80 | 7·25 | 80·39 | 4·26 |
| May 2008 | 2·80 | 7·25 | 80·39 | 4·27 |
| Jun 2008 | 2·80 | 7·25 | 80·39 | 4·24 |
| Jul 2008 | 3·90 | 7·25 | 80·08 | 4·25 |
| Aug 2008 | 3·90 | 7·25 | 80·08 | 4·02 |
| Sep 2008 | 3·90 | 7·00 | 80·08 | 4·29 |
| Oct 2008 | 9·90 | 6·00 | 79·48 | 4·29 |
| Nov 2008 | 9·90 | 5·25 | 79·48 | 4·46 |
| Dec 2008 | 9·90 | 4·25 | 79·48 | 4·57 |
| Jan 2009 | 7·80 | 4·25 | 79·76 | 4·91 |
| Feb 2009 | 7·80 | 3·25 | 79·76 | 5·30 |
| Mar 2009 | 7·80 | 3·25 | 79·76 | 5·71 |
| Apr 2009 | 9·00 | 3·00 | 80·19 | 5·51 |
| May 2009 | 9·00 | 3·00 | 80·19 | 5·81 |
| Jun 2009 | 9·00 | 3·00 | 80·19 | 5·86 |
| Jul 2009 | 5·80 | 3·00 | 81·18 | 5·67 |
| Aug 2009 | 5·80 | 3·00 | 81·18 | 5·71 |
| Sep 2009 | 5·80 | 3·00 | 81·18 | 5·66 |
| Oct 2009 | 5·60 | 3·25 | 82·71 | 5·62 |
| Nov 2009 | 5·60 | 3·50 | 82·71 | 5·56 |
| Dec 2009 | 5·60 | 3·75 | 82·71 | 5·49 |
| Jan 2010 | 6·20 | 3·75 | 83·49 | 5·27 |
| Feb 2010 | 6·20 | 3·75 | 83·49 | 5·30 |
| Mar 2010 | 6·20 | 4·00 | 83·49 | 5·42 |
| Apr 2010 | 5·20 | 4·25 | 83·98 | 5·50 |
| May 2010 | 5·20 | 4·50 | 83·98 | 5·23 |
| Jun 2010 | 5·20 | 4·50 | 83·98 | 5·14 |
| Jul 2010 | 6·30 | 4·50 | 83·67 | 5·29 |
| Aug 2010 | 6·30 | 4·50 | 83·67 | 5·03 |
| Sep 2010 | 6·30 | 4·50 | 83·67 | 5·05 |
| Oct 2010 | 7·80 | 4·50 | 83·14 | 5·28 |
| Nov 2010 | 7·80 | 4·75 | 83·14 | 5·11 |
| Dec 2010 | 7·80 | 4·75 | 83·14 | 4·88 |
| Jan 2011 | 8·40 | 4·75 | 82·49 | 5·00 |
| Feb 2011 | 8·40 | 4·75 | 82·49 | 4·97 |
| Mar 2011 | 8·40 | 4·75 | 82·49 | 4·93 |
| Apr 2011 | 7·80 | 4·75 | 82·05 | 4·96 |
| May 2011 | 7·80 | 4·75 | 82·05 | 5·01 |
| Jun 2011 | 7·80 | 4·75 | 82·05 | 4·92 |
| Jul 2011 | 8·20 | 4·75 | 81·66 | 5·09 |
| Aug 2011 | 8·20 | 4·75 | 81·66 | 5·27 |
| Sep 2011 | 8·20 | 4·75 | 81·66 | 5·22 |
| Oct 2011 | 9·10 | 4·75 | 81·52 | 5·19 |
| Nov 2011 | 9·10 | 4·50 | 81·52 | 5·23 |
| Dec 2011 | 9·10 | 4·25 | 81·52 | 5·20 |
| Jan 2012 | 7·10 | 4·25 | 81·58 | 5·05 |
| Feb 2012 | 7·10 | 4·25 | 81·58 | 5·19 |
| Mar 2012 | 7·10 | 4·25 | 81·58 | 5·18 |
| Apr 2012 | 7·90 | 4·25 | 81·35 | 4·99 |
| May 2012 | 7·90 | 3·75 | 81·35 | 5·20 |
| Jun 2012 | 7·90 | 3·50 | 81·35 | 5·16 |
| Jul 2012 | 5·50 | 3·50 | 81·79 | 5·18 |
| Aug 2012 | 5·50 | 3·50 | 81·79 | 5·16 |
| Sep 2012 | 5·50 | 3·50 | 81·79 | 5·49 |
| Oct 2012 | 6·60 | 3·25 | 82·09 | 5·41 |
| Nov 2012 | 6·60 | 3·25 | 82·09 | 5·26 |
| Dec 2012 | 6·60 | 3·00 | 82·09 | 5·43 |
| Jan 2013 | 7·30 | 3·00 | 82·39 | 5·39 |
| Feb 2013 | 7·30 | 3·00 | 82·39 | 5·39 |
| Mar 2013 | 7·30 | 3·00 | 82·39 | 5·64 |
| Apr 2013 | 7·10 | 3·00 | 82·94 | 5·59 |
| May 2013 | 7·10 | 2·75 | 82·94 | 5·61 |
| Jun 2013 | 7·10 | 2·75 | 82·94 | 5·69 |
| Jul 2013 | 6·80 | 2·75 | 83·01 | 5·63 |
| Aug 2013 | 6·80 | 2·50 | 83·01 | 5·84 |
| Sep 2013 | 6·80 | 2·50 | 83·01 | 5·70 |
| Oct 2013 | 7·60 | 2·50 | 83·05 | 5·82 |
| Nov 2013 | 7·60 | 2·50 | 83·05 | 5·80 |
| Dec 2013 | 7·60 | 2·50 | 83·05 | 5·90 |
| Jan 2014 | 7·50 | 2·50 | 83·23 | 5·95 |
| Feb 2014 | 7·50 | 2·50 | 83·23 | 5·88 |
| Mar 2014 | 7·50 | 2·50 | 83·23 | 5·87 |
| Apr 2014 | 8·30 | 2·50 | 83·35 | 5·80 |
| May 2014 | 8·30 | 2·50 | 83·35 | 5·93 |
| Jun 2014 | 8·30 | 2·50 | 83·35 | 6·05 |
| Jul 2014 | 7·80 | 2·50 | 83·48 | 6·18 |
| Aug 2014 | 7·80 | 2·50 | 83·48 | 6·10 |
| Sep 2014 | 7·80 | 2·50 | 83·48 | 6·24 |
| Oct 2014 | 7·90 | 2·50 | 83·79 | 6·36 |
| Nov 2014 | 7·90 | 2·50 | 83·79 | 6·30 |
| Dec 2014 | 7·90 | 2·50 | 83·79 | 6·11 |
| Jan 2015 | 7·60 | 2·50 | 84·37 | 6·35 |
| Feb 2015 | 7·60 | 2·25 | 84·37 | 6·15 |
| Mar 2015 | 7·60 | 2·25 | 84·37 | 6·10 |
| Apr 2015 | 7·80 | 2·25 | 85·03 | 6·11 |
| May 2015 | 7·80 | 2·00 | 85·03 | 5·93 |
| Jun 2015 | 7·80 | 2·00 | 85·03 | 6·01 |
| Jul 2015 | 7·10 | 2·00 | 86·19 | 6·28 |
| Aug 2015 | 7·10 | 2·00 | 86·19 | 6·09 |
| Sep 2015 | 7·10 | 2·00 | 86·19 | 6·18 |
| Oct 2015 | 5·60 | 2·00 | 88·02 | 5·91 |
| Nov 2015 | 5·60 | 2·00 | 88·02 | 5·85 |
| Dec 2015 | 5·60 | 2·00 | 88·02 | 5·74 |
| Jan 2016 | 5·60 | 2·00 | 89·29 | 6·04 |
| Feb 2016 | 5·60 | 2·00 | 89·29 | 5·69 |
| Mar 2016 | 5·60 | 2·00 | 89·29 | 5·65 |
| Apr 2016 | 6·00 | 2·00 | 90·74 | 5·64 |
| May 2016 | 6·00 | 1·75 | 90·74 | 5·72 |
| Jun 2016 | 6·00 | 1·75 | 90·74 | 5·70 |
| Jul 2016 | 6·00 | 1·75 | 91·94 | 5·70 |
| Aug 2016 | 6·00 | 1·50 | 91·94 | 5·62 |
| Sep 2016 | 6·00 | 1·50 | 91·94 | 5·67 |
| Oct 2016 | 5·50 | 1·50 | 93·11 | 5·60 |
| Nov 2016 | 5·50 | 1·50 | 93·11 | 5·77 |
| Dec 2016 | 5·50 | 1·50 | 93·11 | 5·84 |
| Jan 2017 | 5·60 | 1·50 | 94·53 | 5·68 |
| Feb 2017 | 5·60 | 1·50 | 94·53 | 5·89 |
| Mar 2017 | 5·60 | 1·50 | 94·53 | 5·84 |
| Apr 2017 | 4·90 | 1·50 | 95·94 | 5·60 |
| May 2017 | 4·90 | 1·50 | 95·94 | 5·55 |
| Jun 2017 | 4·90 | 1·50 | 95·94 | 5·62 |
| Jul 2017 | 4·60 | 1·50 | 97·31 | 5·61 |
| Aug 2017 | 4·60 | 1·50 | 97·31 | 5·53 |
| Sep 2017 | 4·60 | 1·50 | 97·31 | 5·47 |
| Oct 2017 | 5·00 | 1·50 | 97·99 | 5·41 |
| Nov 2017 | 5·00 | 1·50 | 97·99 | 5·45 |
| Dec 2017 | 5·00 | 1·50 | 97·99 | 5·62 |
| Jan 2018 | 4·80 | 1·50 | 98·66 | 5·52 |
| Feb 2018 | 4·80 | 1·50 | 98·66 | 5·58 |
| Mar 2018 | 4·80 | 1·50 | 98·66 | 5·53 |
| Apr 2018 | 4·80 | 1·50 | 99·23 | 5·54 |
| May 2018 | 4·80 | 1·50 | 99·23 | 5·44 |
| Jun 2018 | 4·80 | 1·50 | 99·23 | 5·34 |
| Jul 2018 | 5·10 | 1·50 | 99·57 | 5·31 |
| Aug 2018 | 5·10 | 1·50 | 99·57 | 5·25 |
| Sep 2018 | 5·10 | 1·50 | 99·57 | 5·03 |
| Oct 2018 | 5·70 | 1·50 | 99·64 | 5·01 |
| Nov 2018 | 5·70 | 1·50 | 99·64 | 5·11 |
| Dec 2018 | 5·70 | 1·50 | 99·64 | 5·02 |
| Jan 2019 | 6·10 | 1·50 | 99·49 | 5·04 |
| Feb 2019 | 6·10 | 1·50 | 99·49 | 4·98 |
| Mar 2019 | 6·10 | 1·50 | 99·49 | 5·09 |
| Apr 2019 | 5·30 | 1·50 | 99·44 | 5·21 |
| May 2019 | 5·30 | 1·50 | 99·44 | 5·20 |
| Jun 2019 | 5·30 | 1·25 | 99·44 | 5·26 |
| Jul 2019 | 7·60 | 1·00 | 99·29 | 5·29 |
| Aug 2019 | 7·60 | 1·00 | 99·29 | 5·26 |
| Sep 2019 | 7·60 | 1·00 | 99·29 | 5·24 |
| Oct 2019 | 6·70 | 0·75 | 99·80 | 5·31 |
| Nov 2019 | 6·70 | 0·75 | 99·80 | 5·17 |
| Dec 2019 | 6·70 | 0·75 | 99·80 | 5·04 |
| Jan 2020 | 9·40 | 0·75 | 100·18 | 5·24 |
| Feb 2020 | 9·40 | 0·75 | 100·18 | 5·10 |
| Mar 2020 | 9·40 | 0·50 | 100·18 | 5·19 |
| Apr 2020 | 23·90 | 0·25 | 100·24 | 6·29 |
| May 2020 | 23·90 | 0·25 | 100·24 | 6·99 |
| Jun 2020 | 23·90 | 0·25 | 100·24 | 7·43 |
| Jul 2020 | 20·40 | 0·25 | 100·46 | 7·46 |
| Aug 2020 | 20·40 | 0·25 | 100·46 | 6·80 |
| Sep 2020 | 20·40 | 0·25 | 100·46 | 6·95 |
| Oct 2020 | 14·10 | 0·25 | 101·10 | 6·96 |
| Nov 2020 | 14·10 | 0·10 | 101·10 | 6·81 |
| Dec 2020 | 14·10 | 0·10 | 101·10 | 6·55 |
| Jan 2021 | 14·20 | 0·10 | 102·03 | 6·27 |
| Feb 2021 | 14·20 | 0·10 | 102·03 | 5·83 |
| Mar 2021 | 14·20 | 0·10 | 102·03 | 5·65 |
| Apr 2021 | 12·20 | 0·10 | 103·49 | 5·46 |
| May 2021 | 12·20 | 0·10 | 103·49 | 5·10 |
| Jun 2021 | 12·20 | 0·10 | 103·49 | 5·04 |
| Jul 2021 | 20·50 | 0·10 | 104·00 | 4·74 |
| Aug 2021 | 20·50 | 0·10 | 104·00 | 4·58 |
| Sep 2021 | 20·50 | 0·10 | 104·00 | 4·73 |
| Oct 2021 | 13·30 | 0·10 | 104·60 | 5·25 |
| Nov 2021 | 13·30 | 0·10 | 104·60 | 4·63 |
| Dec 2021 | 13·30 | 0·10 | 104·60 | 4·20 |
| Jan 2022 | 11·90 | 0·10 | 104·85 | 4·16 |
| Feb 2022 | 11·90 | 0·10 | 104·85 | 4·02 |
| Mar 2022 | 11·90 | 0·10 | 104·85 | 3·96 |
| Apr 2022 | 8·50 | 0·10 | 104·83 | 3·85 |
| May 2022 | 8·50 | 0·35 | 104·83 | 3·93 |
| Jun 2022 | 8·50 | 0·85 | 104·83 | 3·62 |
| Jul 2022 | 7·30 | 1·35 | 105·10 | 3·46 |
| Aug 2022 | 7·30 | 1·85 | 105·10 | 3·53 |
| Sep 2022 | 7·30 | 2·35 | 105·10 | 3·63 |
| Oct 2022 | 3·50 | 2·60 | 104·99 | 3·45 |
| Nov 2022 | 3·50 | 2·85 | 104·99 | 3·51 |
| Dec 2022 | 3·50 | 3·10 | 104·99 | 3·51 |
| Jan 2023 | 2·30 | 3·10 | 104·93 | 3·66 |
| Feb 2023 | 2·30 | 3·35 | 104·93 | 3·59 |
| Mar 2023 | 2·30 | 3·60 | 104·93 | 3·57 |
| Apr 2023 | 1·80 | 3·60 | 104·33 | 3·70 |
| May 2023 | 1·80 | 3·85 | 104·33 | 3·58 |
| Jun 2023 | 1·80 | 4·10 | 104·33 | 3·54 |
| Jul 2023 | 1·50 | 4·10 | 104·12 | 3·73 |
| Aug 2023 | 1·50 | 4·10 | 104·12 | 3·68 |
| Sep 2023 | 1·50 | 4·10 | 104·12 | 3·60 |
| Oct 2023 | 2·60 | 4·10 | 103·19 | 3·79 |

# **Appendix J. Suicide count and population number by year**

***Table S6.* Suicide count and population number by year**

| **Year** | **Suicide count** | | | | | | **Population number** | | | | | |
| --- | --- | --- | --- | --- | --- | --- | --- | --- | --- | --- | --- | --- |
|  | **All persons** | **Quintile 1** | **Quintile 2** | **Quintile 3** | **Quintile 4** | **Quintile 5** | **All persons** | **Quintile 1** | **Quintile 2** | **Quintile 3** | **Quintile 4** | **Quintile 5** |
| 2006 | 2145 | 507 | 468 | 389 | 411 | 346 | 20448230 | 4222462 | 4034538 | 4100420 | 3900157 | 4190653 |
| 2007 | 2265 | 543 | 438 | 459 | 420 | 393 | 20824802 | 4275394 | 4101097 | 4189693 | 3999643 | 4258975 |
| 2008 | 2328 | 524 | 484 | 515 | 410 | 375 | 21246341 | 4346238 | 4176749 | 4288142 | 4101267 | 4333945 |
| 2009 | 2364 | 557 | 465 | 462 | 457 | 406 | 21688562 | 4420676 | 4254901 | 4389622 | 4205845 | 4417518 |
| 2010 | 2415 | 551 | 505 | 493 | 423 | 416 | 22028504 | 4480594 | 4318292 | 4469250 | 4283888 | 4476480 |
| 2011 | 2478 | 705 | 571 | 453 | 393 | 343 | 22336146 | 4534048 | 4376262 | 4543563 | 4351361 | 4530912 |
| 2012 | 2573 | 708 | 606 | 490 | 400 | 343 | 22729448 | 4585856 | 4447672 | 4641382 | 4452975 | 4601563 |
| 2013 | 2683 | 730 | 576 | 518 | 453 | 369 | 23124098 | 4640158 | 4516272 | 4738873 | 4553399 | 4675396 |
| 2014 | 2909 | 780 | 669 | 528 | 489 | 408 | 23472158 | 4680636 | 4575107 | 4826568 | 4644918 | 4744929 |
| 2015 | 3076 | 821 | 706 | 576 | 501 | 452 | 23812359 | 4718209 | 4628745 | 4912503 | 4738675 | 4814227 |
| 2016 | 2971 | 832 | 697 | 543 | 497 | 378 | 24187198 | 4760007 | 4687276 | 5008292 | 4842433 | 4889190 |
| 2017 | 3350 | 922 | 718 | 649 | 538 | 492 | 24588783 | 4812716 | 4749708 | 5107442 | 4954554 | 4964363 |
| 2018 | 3198 | 875 | 702 | 590 | 518 | 456 | 24959341 | 4858884 | 4805778 | 5201581 | 5063561 | 5029537 |
| 2019 | 3333 | 860 | 788 | 642 | 545 | 452 | 25330776 | 4899815 | 4862242 | 5298684 | 5177563 | 5092472 |
| 2020 | 3193 | 884 | 690 | 596 | 507 | 479 | 25645107 | 4926411 | 4911968 | 5387657 | 5278075 | 5140996 |
| 2021 | 3156 | 899 | 679 | 599 | 521 | 428 | 25681176 | 4909704 | 4916412 | 5429062 | 5316606 | 5109392 |
| 2022 | 3294 | 892 | 729 | 644 | 565 | 429 | 26010017 | 4934755 | 4975390 | 5522822 | 5421821 | 5155229 |
| January-October 2023 | 2644 | 694 | 621 | 495 | 454 | 339 | 26643770 | 5016757 | 5082004 | 5666598 | 5589580 | 5288831 |

# **Appendix K. Joinpoint regression analyses and results**

We estimated change points in monthly suicide rates for all groups using joinpoint models. Monthly suicide rates were used as the dependent variable and month was used the independent variable. The analysis was conducted for all persons and stratified by socioeconomic disadvantage group. Models were fitted with the log-transformed suicide rate as the outcome. Model estimates were transformed to express slopes as the monthly percent change (MPC). For each group, models with zero to seven join points were compared, and the model with the lowest Bayesian Information Criterion was selected as the final model. Monte Carlo Permutation method was used as the test of significance. This analysis was performed using the Joinpoint Trend Analysis Software (version 5·4·0) developed by the National Cancer Institute.^8^

Table S7 shows the results from our joinpoint models. For all persons, there were a statistically significant upward trend in monthly suicide rates from January 2006 to November 2018 (0·16%, 95% CI = 0·13‒0·20, p <0·001), and a statistically significant downward trend from November 2018 to October 2023 (-0·17, 95% CI =-0·40‒-0·04, p = 0·012). For people from Quintile 1 areas, monthly suicide rates increased by 0·38% between January 2006 and March 2017 (95% CI = 0·31‒0·47, p <0·001). For people residing in Quintile 2 areas, monthly suicide rates rose by 0·29% between January 2006 and December 2016 (95% CI = 0·21‒0·44, p <0·001). There was an overall upward trend in monthly suicide rates for people from Quintile 3 areas over the study period (0·05%, 95% CI = 0·01‒0·09, p = 0·014). No evident trend was observed for people from Quintile 4 areas. For people from Quintile 5 areas, monthly suicide rates rose by 0·36% (95% CI = 0·09‒0·35, p = 0·048) between April 2011 and November 2017, and the suicide rates decreased by 0·27% (95% CI = -0·68‒-0·01, p = 0·043) between November 2017 and October 2023.

***Table S7.* Monthly percent change for all groups**

| **Period** | **Monthly percent change** | **95% Confidence Interval** | **P-value** |
| --- | --- | --- | --- |
| **All persons** | | | |
| January 2006‒November 2018 | 0·162 | 0·130‒0·203 | < 0·001* |
| November 2018‒October 2023 | -0·169 | -0·401‒ -0·039 | 0·012* |
| **Quintile 1 (most disadvantage)** | | | |
| January 2006‒March 2017 | 0·377 | 0·311‒0·470 | < 0·001* |
| March 2017‒October 2023 | -0·098 | -0·331‒ 0·042 | 0·168 |
| **Quintile 2** | | | |
| January 2006‒December 2016 | 0·288 | 0·212‒0·435 | < 0·001* |
| December 2016‒October 2023 | -0·078 | -0·447‒0·073 | 0·283 |
| **Quintile 3** | | | |
| January 2006‒October 2023 | 0·050 | 0·010‒0·092 | 0·014* |
| **Quintile 4** | | | |
| January 2006‒October 2023 | -0·002 | -0·039‒0·036 | 0·935 |
| **Quintile 5 (least disadvantage)** | | | |
| January 2006‒January 2011 | 0·190 | -0·134‒0·898 | 0·141 |
| January 2011‒April 2011 | -8·997 | -12·558‒0·127 | 0·052 |
| April 2011‒November 2017 | 0·362 | 0·086‒0·350 | 0·048* |
| November 2017‒October 2023 | -0·267 | -0·678‒-0·010 | 0·043* |

*Significant result (p < 0·05).

^8^National Cancer Institute. Joinpoint Trend Analysis Software (version 5·4·0). <https://surveillance.cancer.gov/joinpoint/> (accessed December 1, 2025)

# **Appendix L. Additional analyses and results**

***Table S8.* Association of (categorical) changes in annual inflation and cash rate (lagged by one month) with monthly suicide rate for all persons and across IRSD quintile groups**

|  | Number of time points | All persons | Quintile 1  (most disadvantage) | Quintile 2 | Quintile 3 | Quintile 4 | Quintile 5  (least disadvantage) |
| --- | --- | --- | --- | --- | --- | --- | --- |
|  | IRR (95% CI) | | | | | | |
| Monthly change in annual inflation in the previous month | | | | | | | |
| ≤0 (reference group) | 97 | 1·00 | 1·00 | 1·00 | 1·00 | 1·00 | 1·00 |
| >0 & <0·2 | 53 | 0·99 (0·96‒1·01,  p = 0·359) | 0·99 (0·94‒1·04,  p = 0·687) | 0·99 (0·94‒1·04,  p = 0·700) | 1·00 (0·95‒1·06,  p = 0·918) | 0·95 (0·90‒1·00,  p = 0·051) | 1·02 (0·95‒1·08,  p = 0·598) |
| ≥0·2 | 62 | 1·03 (1·00‒1·06,  p = 0·037)* | 1·05 (1·00‒1·10,  p = 0·038)* | 0·98 (0·93‒1·03,  p = 0·440) | 1·01 (0·96‒1·07,  p = 0·586) | 1·01 (0·96‒1·06,  p = 0·821) | 1·09 (1·02‒1·15,  p = 0·006)* |
| Monthly change in cash rate in the previous month | | | | | | | |
| <0 | 24 | 0·98 (0·94‒1·01,  p = 0·195) | 0·94 (0·88‒1·00,  p = 0·037)* | 1·01 (0·95‒1·09,  p = 0·671) | 1·04 (0·97‒1·11,  p = 0·230) | 0·94 (0·88‒1·01,  p = 0·090) | 0·97 (0·89‒1·05,  p = 0·466) |
| =0 (reference group) | 162 | 1·00 | 1·00 | 1·00 | 1·00 | 1·00 | 1·00 |
| >0 | 26 | 0·99 (0·95‒1·03,  p = 0·490) | 1·00 (0·94‒1·07,  p = 0·984) | 0·94 (0·88‒1·02,  p = 0·139) | 0·98 (0·91‒1·06,  p = 0·635) | 0·98 (0·92‒1·05,  p = 0·542) | 1·01 (0·92‒1·10,  p = 0·814) |

*Significant result (p < 0·05). All models were adjusted for non-linear time trend, seasonality (if model improved with this variable), monthly change in unemployment rate (lagged by one month), and lagged suicide terms (if needed).

**Macroeconomic changes and suicide rate by sex**

***Table S9.* Adjusted results on the association of stationary macroeconomic factors (lagged by one month/quarter) with suicide rate across sex**

|  | Male | Female |
| --- | --- | --- |
|  | IRR (95% CI) | |
| Monthly change in annual inflation in the previous month | 1·04 (1·00‒1·07,  p = 0·025)* | 1·03 (0·99‒1·08,  p = 0·162) |
| Monthly change in cash rate in the previous month | 1·00 (0·93‒1·07,  p = 0·977) | 1·01 (0·91‒1·13,  p = 0·833) |
| Quarterly change in household saving to income ratio in the previous quarter | 1·00 (0·99‒1·00,  p = 0·537) | 1·00 (0·99‒1·01,  p = 0·758) |
| Quarterly change in owner-occupier housing debt to income ratio in the previous quarter | 0·99 (0·97‒1·02,  p = 0·504) | 1·00 (0·97‒1·04,  p = 0·836) |

*Significant result (p < 0·05). All models were adjusted for non-linear time trend, seasonality (if model improved with this variable), monthly/quarterly change in unemployment rate (lagged by 1 month/quarter), and lagged suicide terms (if needed). We obtained monthly/quarterly suicide data by sex from the ABS. We retrieved quarterly estimated resident population data by sex from 2006 to 2023 from publicly available ABS source, which were subsequently applied to each corresponding quarter to calculate the monthly/quarterly suicide rate for each sex group.

**Macroeconomic changes and suicide rate by age group**

***Table S10*. Adjusted results on the association of stationary macroeconomic factors (lagged by one month/quarter) with suicide rate across age group**

|  | <30 | 30‒49 | 50‒69 | ≥70 |
| --- | --- | --- | --- | --- |
|  | IRR (95% CI) | | | |
| Monthly change in annual inflation in the previous month | 1·04 (0·99‒1·09,  p = 0·094) | 1·01 (0·97‒1·05,  p = 0·601) | 1·01 (0·96‒1·06,  p = 0·655) | 1·09 (1·02‒1·17,  p = 0·011)* |
| Monthly change in cash rate in the previous month | 0·98 (0·87‒1·11,  p = 0·803) | 0·96 (0·88‒1·05,  p = 0·359) | 1·06 (0·94‒1·18,  p = 0·344) | 0·99 (0·84‒1·18,  p = 0·939) |
| Quarterly change in household saving to income ratio in the previous quarter | 1·01 (1·00‒1·01,  p = 0·014)* | 1·00 (0·99‒1·01,  p = 0·820) | 0·99 (0·99‒1·00,  p = 0·110) | 0·99 (0·98‒1·00,  p = 0·228) |
| Quarterly change in owner-occupier housing debt to income ratio in the previous quarter | 0·99 (0·95‒1·02,  p = 0·418) | 1·00 (0·97‒1·04,  p = 0·856) | 1·00 (0·95‒1·04,  p = 0·852) | 0·99 (0·94‒1·05,  p = 0·823) |

*Significant result (p < 0·05). All models were adjusted for non-linear time trend, seasonality (if model improved with this variable), monthly/quarterly change in unemployment rate (lagged by 1 month/quarter), and lagged suicide terms (if needed). We obtained monthly/quarterly suicide data by age group from the ABS. We retrieved quarterly estimated resident population data by age group from 2006 to 2023 from publicly available ABS source, which were subsequently applied to each corresponding quarter to calculate the monthly/quarterly suicide rate for each age group.

# **Appendix M. Sensitivity analyses and results**

We performed 14 sensitivity analyses. We repeated the primary analysis using stationary variables without other adjustments (sensitivity analysis 1), without adjusting for monthly change in unemployment rate (lagged by one month) (sensitivity analysis 2), and with adjustment for the same variables for all groups (non-linear time trend, seasonality, and lagged one to three suicide terms; sensitivity 3). We also repeated the primary analysis using non-lagged macroeconomic indicators (sensitivity analysis 4), macroeconomic indicators lagged by three months (sensitivity analysis 5), and macroeconomic indicators lagged by six months (sensitivity analysis 6). Additionally, we limited our data to the pre-COVID-19 pandemic (2006‒January 2020, sensitivity analysis 7) and during the COVID-19 pandemic and after (February 2020‒October 2023, sensitivity analysis 8). We examined the associations of non-stationary macroeconomic indicators with suicide (unadjusted, sensitivity analysis 9; adjusting for best-fitting variables for each group, sensitivity analysis 10; and adjusting for the same variables for all groups, sensitivity analysis 11). We also repeated the primary analysis using negative binomial regression, adjusting for the same variables for all groups (sensitivity 12). Furthermore, we checked and identified outlier values in exposure variables (those with a z score less than -3 or more than 3) and replaced outlier values with values within the normal range and repeated the primary analysis (sensitivity analysis 13). Finally, we repeated the primary analysis using month rather than quarter (four seasons) to adjust for seasonality (sensitivity analysis 14).

In the models using stationary variables without other adjustments (sensitivity analysis 1; Table S11), one association was not significant (housing debt for Quintile 2 group) and two were marginally not significant (annual inflation for all persons, household savings for Quintile 5 group and). The models without adjustment for monthly/quarterly change in the unemployment rate (lagged by one month/quarter) and the models adjusted for the same variables for all groups showed similar results to our main findings (sensitivity analyses 2 and 3; Table S11).

In the models using non-lagged, lagged by three months, and lagged by six months macroeconomic indicators, several significant associations with suicide rate were not observed (sensitivity analyses 4-6; Tables S12-S14, respectively), indicating that the effect of macroeconomic changes on suicide likely occurred with a 1-month lag rather than immediately or lagged by three or six months (which could be influenced by other factors). Nevertheless, we found that the positive association between housing debt and suicide rate in Quintile 4 areas emerged within the same quarter. The positive association between annual inflation and suicide rate among people residing in the least disadvantaged area also emerged three months later.

When we limited our data to the pre-COVID-19 pandemic period (January 2006‒January 2020), the associations of macroeconomic changes with suicide rate based on our primary analysis mainly only remained in the lesser disadvantaged groups (Quintiles 3‒5; sensitivity analysis 7; Table S15). This implies these groups (wealthier people) were more likely to be affected by the selected macroeconomic changes before the COVID-19 pandemic. On the contrary, when we limited our data to during the COVID-19 pandemic and after (February 2020‒October 2023), several associations disappeared and only monthly change in annual inflation (lagged by one month) was positively associated with suicide in the Quintile 1 group (sensitivity analysis 8; Table S16). However, these findings (based on 45 time points) could be the result of a lack of statistical power to detect significant associations.

The findings from our sensitivity analyses using non-stationary macroeconomic indicators, from both unadjusted and adjusted models, were substantially different from those from our primary analyses using stationary indicators (sensitivity analyses 9‒11; Tables S17). The results changed substantially (even showing a change in direction) between the unadjusted models, models adjusted for the best-fitting variables, and those adjusted for the same set of variables. This contrasted with the models using stationary macroeconomic indicators, for which the results of the models across alternative model specifications were generally similar. These indicate that non-stationarity has seriously biased the results as the IRRs of non-stationary predictors are likely to be driven by underlying trends rather than reflect true associations.

When we used negative binomial regression, adjusting for the same variables for all groups (sensitivity analysis 12, Table S18), the results were generally consistent with those from the primary analysis. In these models, a quarterly change in housing debt to income ratio (lagged by one quarter) was also associated with an increase in quarterly suicide rate for Quintile 4 group.

In our sensitivity analysis 13 (Table S19) where we replaced outlier values in macroeconomic changes with values within the normal range, the association between cash rate and suicide rates for Quintile 3 group became marginally non-significant. We also did not observe an association between housing debt and suicide for Quintile 2 group.

When we used month instead of season to adjust for seasonality (Table S20), only the negative association between cash rate and suicide rates for Quintile 3 areas became marginally non-significant. All other results remained the same as those from our primary analysis.

The findings from these sensitivity analyses suggest that the models used in our primary analyses are robust, as the estimates remained generally consistent across alternative model specifications (adjusting for different variables and using negative binomial regression).

***Table S11.* Unadjusted and adjusted (different variables) results on the association of *stationary* macroeconomic factors (lagged by one month/quarter) with suicide rate for all persons and across IRSD quintile groups (sensitivity analyses 1‒3)**

|  | All persons | Quintile 1  (most disadvantage) | Quintile 2 | Quintile 3 | Quintile 4 | Quintile 5  (least disadvantage) |
| --- | --- | --- | --- | --- | --- | --- |
|  | IRR (95% CI) | | | | | |
| Sensitivity analysis 1: unadjusted models | | | | | | |
| Monthly change in annual inflation in the previous month  (Models 1a by group) | 1·03 (1·00‒1·07,  p = 0·091) | 1·08 (1·02‒1·15,  p = 0·013)* | 0·99 (0·93‒1·05,  p = 0·738) | 1·01 (0·96‒1·06,  p = 0·693) | 0·99 (0·94‒1·04,  p = 0·580) | 1·08 (1·02‒1·14,  p = 0·013)* |
| Monthly change in cash rate in the previous month  (Models 1b by group) | 0·99 (0·91‒1·08,  p = 0·838) | 1·10 (0·95‒1·28,  p = 0·213) | 0·95 (0·82‒1·09,  p = 0·451) | 0·88 (0·78‒0·99,  p = 0·032)* | 1·04 (0·92‒1·17,  p = 0·537) | 1·01 (0·88‒1·16,  p = 0·853) |
| Quarterly change in household saving to income ratio in the previous quarter (Models 1c by group) | 1·00 (0·99‒1·01,  p = 0·596) | 0·99 (0·98‒1·01,  p = 0·354) | 0·99 (0·98‒1·01,  p = 0·243) | 1·00 (0·99‒1·01,  p = 0·629) | 1·00 (0·99‒1·00,  p = 0·370) | 1·01 (1·00‒1·02,  p = 0·072) |
| Quarterly change in owner-occupier housing debt to income ratio in the previous quarter(Models 1d by group) | 1·03 (0·99‒1·07,  p = 0·130) | 1·04 (0·98‒1·12,  p = 0·206) | 1·02 (0·96‒1·08,  p = 0·539) | 1·01 (0·97‒1·06,  p = 0·616) | 1·04 (1·01‒1·08,  p = 0·023)* | 1·03 (0·99‒1·08,  p = 0·165) |
| Sensitivity analysis 2: models adjusted for non-linear time trend, seasonality (if model improved with this variable), and lagged suicide terms (if needed) | | | | | | |
| Monthly change in annual inflation in the previous month  (Models 2a by group) | 1·03 (1·00‒1·06,  p = 0·022)* | 1·07 (1·02‒1·11,  p = 0·004)* | 0·98 (0·93‒1·03,  p = 0·415) | 1·00 (0·96‒1·05,  p = 0·888) | 0·99 (0·94‒1·04,  p = 0·573) | 1·08 (1·02‒1·14,  p = 0·006)* |
| Monthly change in cash rate in the previous month  (Models 2b by group) | 1·00 (0·94‒1·07,  p = 0·926) | 1·08 (0·96‒1·21,  p = 0·202) | 0·92 (0·82‒1·04,  p = 0·204) | 0·88 (0·78‒0·99,  p = 0·032)* | 1·04 (0·92‒1·17,  p = 0·526) | 1·06 (0·92‒1·21,  p = 0·429) |
| Quarterly change in household saving to income ratio in the previous quarter (Models 2c by group) | 1·00 (1·00‒1·00,  p = 0·552) | 1·00 (0·99‒1·00,  p = 0·362) | 1·00 (0·99‒1·00,  p = 0·173) | 1·00 (0·99‒1·01,  p = 0·482) | 1·00 (0·99‒1·01,  p = 0·609) | 1·01 (1·00‒1·02,  p = 0·013)* |
| Quarterly change in owner-occupier housing debt to income ratio in the previous quarter  (Models 2d by group) | 0·99 (0·97‒1·01,  p = 0·417) | 0·98 (0·95‒1·02,  p = 0·307) | 0·97 (0·94‒1·00,  p = 0·056) | 1·02 (0·98‒1·06,  p = 0·296) | 1·04 (1·00‒1·08,  p = 0·059) | 1·01 (0·96‒1·06,  p = 0·658) |
| Sensitivity analysis 3: models adjusted for the same variables (non-linear time trend, seasonality, and lagged one to three suicide terms) and monthly/quarterly change in unemployment rate (lagged by 1 month/quarter) | | | | | | |
| Monthly change in annual inflation in the previous month  (Models 3a by group) | 1·03 (1·00‒1·06,  p = 0·032)* | 1·08 (1·03‒1·13,  p = 0·001)* | 0·98 (0·93‒1·03,  p = 0·360) | 1·01 (0·96‒1·06,  p = 0·807) | 0·99 (0·94‒1·04,  p = 0·722) | 1·07 (1·01‒1·14,  p = 0·023)* |
| Monthly change in cash rate in the previous month  (Models 3b by group) | 0·99 (0·93‒1·06,  p = 0·766) | 1·09 (0·97‒1·22,  p = 0·139) | 0·92 (0·81‒1·04,  p = 0·184) | 0·87 (0·77‒0·99,  p = 0·028)* | 1·03 (0·91‒1·17,  p = 0·631) | 1·03 (0·89‒1·20,  p = 0·667) |
| Quarterly change in household saving to income ratio in the previous quarter (Models 3c by group) | 1·00 (1·00‒1·00,  p = 0·800) | 1·00 (0·99‒1·00,  p = 0·398) | 1·00 (0·99‒1·00,  p = 0·177) | 1·00 (0·99‒1·01,  p = 0·540) | 1·00 (0·99‒1·01,  p = 0·713) | 1·01 (1·00‒1·02,  p = 0·019)* |
| Quarterly change in owner-occupier housing debt to income ratio in the previous quarter  (Models 3d by group) | 0·99 (0·97‒1·01,  p = 0·519) | 0.97 (0·94‒1·01,  p = 0·161) | 0·97 (0·93‒1·00,  p = 0·047)* | 1·02 (0·98‒1·07,  p = 0·248) | 1·03 (0·99‒1·08,  p = 0·093) | 1·02 (0·97‒1·07,  p = 0·352) |

*Significant result (p < 0·05).

***Table S12.* Adjusted results on the association of *stationary* macroeconomic factors (non-lagged) with suicide rate for all persons and across IRSD quintile groups (sensitivity analysis 4)**

| Sensitivity analysis 4 | All persons | Quintile 1  (most disadvantage) | Quintile 2 | Quintile 3 | Quintile 4 | Quintile 5  (least disadvantage) |
| --- | --- | --- | --- | --- | --- | --- |
|  | IRR (95% CI) in the same month | | | | | |
| Monthly change in annual inflation  (Models 4a by group) | 1·00 (0·97‒1·03,  p = 0·868) | 0·98 (0·94‒1·03,  p = 0·476) | 1·01 (0·96‒1·06,  p = 0·786) | 1·02 (0·97‒1·08,  p = 0·374) | 1·01 (0·96‒1·06,  p = 0·818) | 0·95 (0·90‒1·01,  p = 0·119) |
| Monthly change in cash rate  (Models 4b by group) | 0·99 (0·93‒1·06,  p = 0·870) | 1·00 (0·89‒1·12,  p = 0·990) | 1·02 (0·90‒1·16,  p = 0·708) | 0·92 (0·82‒1·04,  p = 0·175) | 1·03 (0·91‒1·16,  p = 0·668) | 1·03 (0·89‒1·19,  p = 0·725) |
| Quarterly change in household saving to income ratio  (Models 4c by group) | 1·00 (0·99‒1·00,  p = 0·116) | 1·00 (0·99‒1·00,  p = 0·2303) | 1·00 (0·99‒1·00,  p = 0·258) | 1·00 (1·00‒1·01,  p = 0·396) | 1·00 (0·99‒1·00,  p = 0·405) | 0·99 (0·98‒1·00,  p = 0·093) |
| Quarterly change in owner-occupier housing debt to income ratio  (Models 4d by group) | 1·00 (0·98‒1·02,  p = 0·963) | 1·00 (0·96‒1·04,  p = 0·965) | 0·99 (0·95‒1·02,  p = 0·504) | 1·00 (0·96‒1·04,  p = 0·956) | 1·06 (1·02‒1·10,  p = 0·004)* | 1·03 (0·99‒1·09,  p = 0·175) |

*Significant result (p < 0·05). All models were adjusted for non-linear time trend, seasonality (if model improved with this variable), monthly/quarterly change in unemployment rate (non-lagged), and lagged suicide terms (if needed).

***Table S13.* Adjusted results on the association of *stationary* macroeconomic factors (lagged by three months) with suicide rate for all persons and across IRSD quintile groups sensitivity analysis 5)**

| Sensitivity analysis 5 | All persons | Quintile 1  (most disadvantage) | Quintile 2 | Quintile 3 | Quintile 4 | Quintile 5  (least disadvantage) |
| --- | --- | --- | --- | --- | --- | --- |
|  | IRR (95% CI) | | | | | |
| Monthly change in annual inflation occurred three months ago  (Models 5a by group) | 1·00 (0·97‒1·03,  p = 0·831) | 1·00 (0·96‒1·05,  p = 0·900) | 0·99 (0·94‒1·04,  p = 0·658) | 0·97 (0·92‒1·02,  p = 0·203) | 1·03 (0·98‒1·09,  p = 0·234) | 1·07 (1·00‒1·13,  p = 0·043)* |
| Monthly change in cash rate occurred three months ago  (Models 5b by group) | 1·00 (0·94‒1·06,  p = 0·945) | 0·94 (0·84‒1·05,  p = 0·295) | 0·96 (0·84‒1·09,  p = 0·499) | 1·03 (0·91‒1·17,  p = 0·626) | 1·02 (0·91‒1·15,  p = 0·697) | 1·00 (0·86‒1·16,  p = 0·984) |

*Significant result (p < 0·05). All models were adjusted for non-linear time trend, seasonality (if model improved with this variable), monthly change in unemployment rate (lagged by three months), and lagged suicide terms (if needed).

***Table S14.* Adjusted results on the association of *stationary* macroeconomic factors (lagged by six months/two quarters) with suicide rate for all persons and across IRSD quintile groups sensitivity analysis 6)**

| Sensitivity analysis 6 8 | All persons | Quintile 1  (most disadvantage) | Quintile 2 | Quintile 3 | Quintile 4 | Quintile 5  (least disadvantage) |
| --- | --- | --- | --- | --- | --- | --- |
|  | IRR (95% CI) | | | | | |
| Monthly change in annual inflation occurred six months ago  (Models 6a by group) | 1·04 (1·01‒1·06,  p = 0·014)* | 1·03 (0·99‒1·08,  p = 0·167) | 0·99 (0·94‒1·05,  p = 0·797) | 1·05 (1·00‒1·11,  p = 0·042)* | 1·01 (0·96‒1·07,  p = 0·583) | 1·02 (0·96‒1·08,  p = 0·584) |
| Monthly change in cash rate occurred six months ago  (Models 6b by group) | 1·01 (0·95‒1·08,  p = 0·728) | 1·04 (0·92‒1·16,  p = 0·536) | 1·02 (0·90‒1·16,  p = 0·752) | 1·17 (1·03‒1·33,  p = 0·015)* | 0·91 (0·81‒1·03,  p = 0·129) | 0·95 (0·82‒1·10,  p = 0·496) |
| Quarterly change in household saving to income ratio occurred two quarters ago  (Models 6c by group) | 1·00 (0·99‒1·00,  p = 0·499) | 1·00 (0·99‒1·00,  p = 0·471) | 1·00 (0·99‒1·01,  p = 0·676) | 1·00 (0·99‒1·01,  p = 0·720) | 1·00 (0·99‒1·01,  p = 0·488) | 1·00 (0·99‒1·01,  p = 0·868) |
| Quarterly change in owner-occupier housing debt to income ratio occurred two quarters ago  (Models 6d by group) | 0·99 (0·97‒1·01,  p = 0·426) | 0·98 (0·95‒1·02,  p = 0·337) | 0·96 (0·93‒0·99,  p = 0·017)* | 1·03 (0·98‒1·07,  p = 0·221) | 1·03 (0·99‒1·07,  p = 0·201) | 1·02 (0·97‒1·07,  p = 0·447) |

*Significant result (p < 0·05). All models were adjusted for non-linear time trend, seasonality (if model improved with this variable), monthly/quarterly change in unemployment rate (lagged by six months/two quarters), and lagged suicide terms (if needed).

***Table S15.* Adjusted results on the association of *stationary* macroeconomic factors (lagged by one month/quarter) with suicide rate for all persons and across IRSD quintile groups from 2006 to January 2020 (pre-COVID-19 pandemic) (sensitivity analysis 7)**

| Sensitivity analysis 7 | All persons | Quintile 1  (most disadvantage) | Quintile 2 | Quintile 3 | Quintile 4 | Quintile 5  (least disadvantage) |
| --- | --- | --- | --- | --- | --- | --- |
|  | IRR (95% CI) | | | | | |
| Monthly change in annual inflation in the previous month  (Models 7a by group) | 1·01 (0·97‒1·05,  p = 0·707) | 1·02 (0·95‒1·08,  p = 0·638) | 0·97 (0·90‒1·05,  p = 0·496) | 0·98 (0·91‒1·05,  p = 0·602) | 0·98 (0·91‒1·06,  p = 0·639) | 1·09 (1·00‒1·19,  p = 0·047)* |
| Monthly change in cash rate in the previous month  (Models 7b by group) | 0·96 (0·89‒1·03,  p = 0·206) | 1·06 (0·94‒1·21,  p = 0·339) | 0·88 (0·76‒1·02,  p = 0·089) | 0·84 (0·74‒0·96,  p = 0·009)* | 1·02 (0·89‒1·18,  p = 0·769) | 1·05 (0·89‒1·23,  p = 0·599) |
| Quarterly change in household saving to income ratio in the previous quarter  (Models 7c by group) | 1·01 (1·00‒1·02,  p = 0·164) | 1·00 (0·98‒1·02,  p = 0·736) | 1·01 (0·99‒1·03,  p = 0·246) | 1·01 (0·99‒1·03,  p = 0·526) | 1·00 (0·98‒1·02,  p = 0·709) | 1·01 (0·99‒1·04,  p = 0·226) |
| Quarterly change in owner-occupier housing debt to income ratio in the previous quarter  (Models 7d by group) | 1·00 (0·97‒1·02,  p = 0·673) | 0·97 (0·94‒1·02,  p = 0·229) | 0·97 (0·93‒1·00,  p = 0·057) | 1·02 (0·97‒1·07,  p = 0·476) | 1·05 (1·01‒1·10,  p = 0·019)* | 1·04 (0·99‒1·10,  p = 0·097) |

*Significant result (p < 0·05). All models were adjusted for non-linear time trend, seasonality (if model improved with this variable), monthly change in unemployment rate (lagged by 1 month), and lagged suicide terms (if needed). For analyses at the quarter level, time points from second quarter of 2020 were dropped.

***Table S16.* Adjusted results on the association of stationary macroeconomic factors (lagged by one month/quarter) with suicide rate for all persons and across IRSD quintile groups from February 2020 to October 2023 (during and post COVID-19 pandemic) (sensitivity analysis 8)**

| Sensitivity analysis 8 | All persons | Quintile 1  (most disadvantage) | Quintile 2 | Quintile 3 | Quintile 4 | Quintile 5  (least disadvantage) |
| --- | --- | --- | --- | --- | --- | --- |
|  | IRR (95% CI) | | | | | |
| Monthly change in annual inflation in the previous month  (Models 8a by group) | 1·04 (0·96‒1·12,  p = 0·322) | 1·12 (1·03‒1·23,  p = 0·012)* | 0·97 (0·89‒1·06,  p = 0·511) | 1·04 (0·95‒1·15,  p = 0·380) | 0·99 (0·89‒1·09,  p = 0·769) | 1·04 (0·94‒1·16,  p = 0·447) |
| Monthly change in cash rate in the previous month  (Models 8b by group) | 0·91 (0·65‒1·27,  p = 0·571) | 0·96 (0·65‒1·43,  p = 0·857) | 0·84 (0·59‒1·20,  p = 0·337) | 0·88 (0·59‒1·32,  p = 0·544) | 1·13 (0·76‒1·66,  p = 0·550) | 1·11 (0·71‒1·73,  p = 0·662) |

*Significant result (p < 0·05). Models were adjusted for non-linear time trend, seasonality (if model improved with this variable), monthly/quarterly change in unemployment rate (lagged by 1 month), and lagged suicide terms (if needed). Analyses at the quarter level for household saving to income ratio and housing debt to income ratio were not conducted due to very small number of time points (n = 14).

***Table S17.* Unadjusted and adjusted results on the association of non-stationary macroeconomic factors (lagged by one month/quarter) with suicide rate for all persons and across IRSD quintile groups (sensitivity analyses 9‒11)**

|  | All persons | Quintile 1  (most disadvantage) | Quintile 2 | Quintile 3 | Quintile 4 | Quintile 5  (least disadvantage) |
| --- | --- | --- | --- | --- | --- | --- |
|  | IRR (95% CI) | | | | | |
| Sensitivity analysis 9: unadjusted models | | | | | | |
| Annual inflation in the previous month  (Models 9a by group) | 0·99 (0·98‒1·00,  p = 0·124) | 0·99 (0·97‒1·01,  p = 0·225) | 0·99 (0·98‒1·01,  p = 0·469) | 1·00 (0·98‒1·01,  p = 0·790) | 1·00 (0·98‒1·01,  p = 0·540) | 0·99 (0·97‒1·00,  p = 0·092) |
| Cash rate in the previous month  (Models 9b by group) | 0·97 (0·96‒0·98,  p < 0·001)* | 0·94 (0·93‒0·95,  p < 0·001)* | 0·96 (0·95‒0·97,  p < 0·001)* | 0·99 (0·98‒1·00,  p = 0·024)* | 1·00 (0·99‒1·01,  p = 0·564) | 0·99 (0·98‒1·00,  p = 0·148) |
| Household saving to income ratio in the previous quarter  (Models 9c by group) | 1·01 (1·00‒1·01,  p = 0·024)* | 1·01 (1·01‒1·02,  p < 0·001)* | 1·01 (1·00‒1·01,  p = 0·075) | 1·00 (1·00‒1·01,  p = 0·470) | 1·00 (0·99‒1·00,  p = 0·239) | 1·00 (1·00‒1·01,  p = 0·416) |
| Owner-occupier housing debt to income ratio in the previous quarter  (Models 9d by group) | 1·01 (1·00‒1·01,  p < 0·001)* | 1·01 (1·01‒1·02,  p < 0·001)* | 1·01 (1·00‒1·01,  p < 0·0001)* | 1·00 (1·00‒1·01,  p = 0·041)* | 1·00 (1·00‒1·00,  p = 0·982) | 1·00 (1·00‒1·00,  p = 0·572) |
| Sensitivity analysis 10: all models adjusted for non-linear time trend, seasonality (if model improved with this variable), monthly/quarterly change in unemployment rate (lagged by 1 month/quarter), and lagged suicide terms (if needed) | | | | | | |
| Annual inflation in the previous month  (Models 10a by group) | 1·01 (1·00‒1·03,  p = 0·061) | 1·02 (1·00‒1·04,  p = 0·101) | 1·00 (0·98‒1·03,  p = 0·739) | 1·01 (0·98‒1·04,  p = 0·546) | 1·01 (0·98‒1·03,  p = 0·563) | 1·01 (0·98‒1·04,  p = 0·572) |
| Cash rate in the previous month  (Models 10b by group) | 1·01 (0·99‒1·02,  p = 0·475) | 1·01 (0·98‒1·03,  p = 0·615) | 1·02 (0·99‒1·05,  p = 0·189) | 1·01 (0·98‒1·04,  p = 0·548) | 0·99 (0·98‒1·01,  p = 0·301) | 0·99 (0·96‒1·03,  p = 0·713) |
| Household saving to income ratio in the previous quarter  (Models 10c by group) | 1·00 (0·99‒1·00,  p = 0·254) | 1·00 (0·99‒1·01,  p = 0·905) | 1·00 (0·99‒1·00,  p = 0·119) | 1·00 (0·99‒1·00,  p = 0·496) | 1·00 (0·99‒1·00,  p = 0·247) | 1·00 (0·99‒1·01,  p = 0·796) |
| Owner-occupier housing debt to income ratio in the previous quarter  (Models 10d by group) | 1·00 (0·99‒1·00,  p = 0·170) | 1·00 (0·99‒1·00,  p = 0·340) | 0·99 (0·98‒1·00,  p = 0·133) | 1·00 (1·00‒1·01,  p = 0·104) | 1·00 (1·00‒1·01,  p = 0·054) | 1·01 (1·00‒1·03,  p = 0·070) |
| Sensitivity analysis 11: all models adjusted for same variables (non-linear time trend, seasonality, and lagged one to three suicide terms) | | | | | | |
| Annual inflation in the previous month  (Models 11a by group) | 1·01 (1·00‒1·02,  p = 0·047)* | 1·02 (1·00‒1·03,  p = 0·072) | 1·02 (1·00‒1·04,  p = 0·061) | 1·02 (1·00‒1·04,  p = 0·068) | 0·99 (0·98‒1·01,  p = 0·246) | 1·00 (0·97‒1·02,  p = 0·916) |
| Cash rate in the previous month  (Models 11b by group) | 1·01 (0·99‒1·02,  p = 0·334) | 1·01 (0·99‒1·03,  p = 0·352) | 1·03 (1·00‒1·06,  p = 0·021)* | 1·02 (1·00‒1·05,  p = 0·112) | 0·98 (0·96‒1·00,  p = 0·017)* | 0·99 (0·96‒1·02,  p = 0·457) |
| Household saving to income ratio in the previous quarter (Models 11c by group) | 1·00 (1·00‒1·00,  p = 0·394) | 1·00 (0·99‒1·01,  p = 0·912) | 1·00 (0·99‒1·00,  p = 0·119) | 1·00 (0·99‒1·00,  p = 0·414) | 1·00 (0·99‒1·00,  p = 0·324) | 1·00 (0·99‒1·01,  p = 0·796) |
| Owner-occupier housing debt to income ratio in the previous quarter (Models 11d by group) | 1·00 (0·99‒1·00,  p = 0·240) | 1·00 (0·99‒1·00,  p = 0·343) | 0·99 (0·98‒1·00,  p = 0·133) | 1·00 (1·00‒1·01,  p = 0·165) | 1·00 (1·00‒1·01,  p = 0·501) | 1·01 (1·00‒1·03,  p = 0·070) |

*Significant result (p < 0·05).

***Table S18.* Adjusted results on the association of *stationary* macroeconomic factors (lagged by one month/quarter) with suicide rate for all persons and across IRSD quintile groups using negative binomial regression model (sensitivity analysis 12)**

| Sensitivity analysis 12 | All persons | Quintile 1  (most disadvantage) | Quintile 2 | Quintile 3 | Quintile 4 | Quintile 5  (least disadvantage) |
| --- | --- | --- | --- | --- | --- | --- |
|  | IRR (95% CI) | | | | | |
| Monthly change in annual inflation in the previous month (Models 12a by group) | 1·03 (1·01‒1·05,  p = 0·013)* | 1·08 (1·03‒1·13,  p = 0·001)* | 0·98 (0·93‒1·02,  p = 0·310) | 1·01 (0·96‒1·05,  p = 0·782) | 0·99 (0·95‒1·03,  p = 0·667) | 1·07 (1·02‒1·13,  p = 0·010)* |
| Monthly change in cash rate in the previous month  (Models 12b by group) | 0·99 (0·94‒1·04,  p = 0·642) | 1·09 (1·00‒1·19,  p = 0·060) | 0·92 (0·82‒1·02,  p = 0·115) | 0·87 (0·78‒0·98,  p = 0·020)* | 1·03 (0·92‒1·16,  p = 0·619) | 1·03 (0·87‒1·23,  p = 0·719) |
| Quarterly change in household saving to income ratio in the previous quarter (Models 12c by group) | 1·00 (1·00‒1·00,  p = 0·759) | 1·00 (0·99‒1·00,  p = 0·087) | 1·00 (0·99‒1·00,  p = 0·173) | 1·00 (1·00‒1·01,  p = 0·375) | 1·00 (0·99‒1·00,  p = 0·612) | 1·01 (1·01‒1·02,  p < 0·001)* |
| Quarterly change in owner-occupier housing debt to income ratio in the previous quarter  (Models 12d by group) | 0·99 (0·97‒1·01,  p = 0·470) | 0·97 (0·94‒1·01,  p = 0·191) | 0·97 (0·94‒0·99,  p = 0·011)* | 1·02 (0·98‒1·07,  p = 0·244) | 1·03 (1·00‒1·07,  p = 0·040)* | 1·02 (0·97‒1·07,  p = 0·361) |

*Significant result (p < 0·05). Models adjusted for the same variables (non-linear time trend, seasonality, and lagged one to three suicide terms) and monthly/quarterly change in unemployment rate (lagged by 1 month/quarter).

***Table S19.* Adjusted results on the association of *stationary* macroeconomic factors (lagged by one month/quarter) with suicide rate for all persons and across IRSD quintile groups (replacing outliers with values within the normal range, sensitivity analysis 13)**

| Sensitivity analysis 13 | All persons | Quintile 1  (most disadvantage) | Quintile 2 | Quintile 3 | Quintile 4 | Quintile 5  (least disadvantage) |
| --- | --- | --- | --- | --- | --- | --- |
|  | IRR (95% CI) | | | | | |
| Monthly change in annual inflation in the previous month  (Models 13a by group) | 1·04 (1·01‒1·07,  p = 0·017)* | 1·08 (1·03‒1·13,  p = 0·002)* | 0·97 (0·92‒1·03,  p = 0·347) | 1·00 (0·95‒1·06,  p = 0·860) | 0·99 (0·93‒1·04,  p = 0·610) | 1·08 (1·01‒1·16,  p = 0·018)* |
| Monthly change in cash rate in the previous month  (Models 13b by group) | 1·03 (0·95‒1·11,  p = 0·518) | 1·12 (0·98‒1·29,  p = 0·105) | 0·91 (0·78‒1·07,  p = 0·247) | 0·88 (0·75‒1·02,  p = 0·085) | 1·09 (0·94‒1·26,  p = 0·254) | 1·08 (0·89‒1·30,  p = 0·429) |
| Monthly change in household saving to income ratio in the previous month (Models 13c by group) | 1·00 (0·99‒1·00,  p = 0·686) | 1·00 (0·99‒1·00,  p = 0·398) | 1·00 (0·99‒1·00,  p = 0·341) | 1·00 (0·99‒1·01,  p = 0·534) | 1·00 (0·99‒1·01,  p = 0·662) | 1·01 (1·00‒1·02,  p = 0·026)* |

*Significant result (p < 0·05). Models were adjusted for non-linear time trend, seasonality (if model improved with this variable), monthly/quarterly change in unemployment rate (lagged by 1 month/quarter), and lagged suicide terms (if needed). The following outlier values were identified: April 2020 and April 2021 for annual inflation; October 2008, November 2008, December 2008, and February 2009 for cash rate; and 2020 second quarter for household saving to income ratio. No outlier was identified for housing debt to income ratio; therefore, this sensitivity analysis was not conducted for this variable.

***Table S20.* Adjusted results on the association of *stationary* macroeconomic factors (lagged by one month) with suicide rate for all persons and across IRSD quintile groups using month rather than quarter to adjust for seasonality (sensitivity analysis 14)**

| Sensitivity analysis 14 | All persons | Quintile 1  (most disadvantage) | Quintile 2 | Quintile 3 | Quintile 4 | Quintile 5  (least disadvantage) |
| --- | --- | --- | --- | --- | --- | --- |
|  | IRR (95% CI) | | | | | |
| Monthly change in annual inflation in the previous month  (Models 14a by group) | 1·03 (1·01‒1·06,  p = 0·014)* | 1·08 (1·03‒1·12,  p = 0·001)* | 0·98 (0·93‒1·03,  p = 0·347) | 1·00 (0·95‒1·05,  p = 0·933) | 0·99 (0·94‒1·04,  p = 0·739) | 1·07 (1·01‒1·14,  p = 0·026)* |
| Monthly change in cash rate in the previous month  (Models 14b by group) | 1·01 (0·95‒1·06,  p = 0·837) | 1·11 (0·99‒1·24,  p = 0·066) | 0·94 (0·84‒1·06,  p = 0·309) | 0·90 (0·80‒1·00,  p = 0·061) | 1·05 (0·93‒1·19,  p = 0·412) | 1·04 (0·89‒1·20,  p = 0·637) |

*Significant result (p < 0·05). Models were adjusted for non-linear time trend, seasonality (if model improved with this variable), monthly change in unemployment rate (lagged by 1 month), and lagged suicide terms (if needed).

# Appendix N. Supplementary figures

***Figure S1.* Monthly/quarterly macroeconomic factors and monthly suicide rate for Quintile 1 areas**

***Figure S2.* Monthly/quarterly macroeconomic factors and monthly suicide rate for Quintile 2 areas**

***Figure S3.* Monthly/quarterly macroeconomic factors and monthly suicide rate for Quintile 3 areas**

***Figure S4.* Monthly/quarterly macroeconomic factors and monthly suicide rate for Quintile 4 areas**

***Figure S5.* Monthly/quarterly macroeconomic factors and monthly suicide rate for Quintile 5 areas**

# Appendix O. Proportion of dwellings by IRSD group across region

*Table S22.* Proportion of dwellings by IRSD group across region (based on 2021 ABS Census data)

| **Region** | **Quintile 1** | **Quintile 2** | **Quintile 3** | **Quintile 4** | **Quintile 5** |
| --- | --- | --- | --- | --- | --- |
| NSW | 20·01 | 21·54 | 20·14 | 15·86 | 22·45 |
| Victoria | 13·92 | 18·92 | 21·39 | 22·59 | 23·18 |
| Queensland | 23·11 | 19·25 | 22·77 | 22·52 | 12·34 |
| South Australia | 25·65 | 26·24 | 21·25 | 16·78 | 10·07 |
| Western Australia | 13·70 | 20·37 | 24·04 | 22·54 | 19·33 |
| Tasmania | 41·05 | 17·07 | 23·51 | 15·77 | 2·60 |
| Northern Territory | 25·72 | 26·10 | 7·66 | 27·94 | 12·46 |
| Australian Capital Territory | 0·33 | 0·70 | 4·67 | 40·72 | 53·52 |

# Appendix P. Data handling procedures

PCFL and LST obtained monthly data from the data sources (see Appendices B and D) in excel format. There were no missing data for suicide and all macroeconomic exposures. As indicated in Appendix B, data cells with small suicide counts were randomly assigned to protect the confidentiality of individuals, which result in some totals not equalling the sum of their components. Cells with a zero value were not affected by this confidentiality procedure.

SA2-level annual estimated resident populations (2006-2023) and SA2-level IRSD (based on 2021 Census data) were retrieved from the ABS. These data were merged to obtain annual population number by IRSD quintile groups, which were then applied to each corresponding year to estimate the monthly suicide rate for each group. Around 4% (101/2454) of SA2 areas were not assigned a decile score, either due to small populations or poor data quality. These SA2 areas were excluded from our analyses by quintile groups but were included in analyses for all persons.

PCFL combined all monthly data by socioeconomic disadvantage groups in Stata. The resulting database was checked by LST to ensure data accuracy and consistency prior to conducting analyses. The Stata code used to generate the main findings is presented in Appendix H.
